# Non-doped hot-exciton blue organic light-emitting diodes with efficiency over 20%

**DOI:** 10.1093/nsr/nwag056

**Published:** 2026-01-31

**Authors:** Mingke Li, Yulong Li, Yue Yu, Yichao Chen, Jianhui Pan, Feng Peng, Dezhi Yang, Dongge Ma, Lei Ying, Yuguang Ma

**Affiliations:** Institute of Polymer Optoelectronic Materials and Devices, Guangdong Basic Research Center of Excellence for Energy and Information Polymer Materials, State Key Laboratory of Luminescent Materials and Devices, South China University of Technology, Guangzhou 510640, China; Institute of Polymer Optoelectronic Materials and Devices, Guangdong Basic Research Center of Excellence for Energy and Information Polymer Materials, State Key Laboratory of Luminescent Materials and Devices, South China University of Technology, Guangzhou 510640, China; Institute of Polymer Optoelectronic Materials and Devices, Guangdong Basic Research Center of Excellence for Energy and Information Polymer Materials, State Key Laboratory of Luminescent Materials and Devices, South China University of Technology, Guangzhou 510640, China; Dongguan Volt-Amp Optoelectronics Technology Co., Ltd., Dongguan 523000, China; Institute of Polymer Optoelectronic Materials and Devices, Guangdong Basic Research Center of Excellence for Energy and Information Polymer Materials, State Key Laboratory of Luminescent Materials and Devices, South China University of Technology, Guangzhou 510640, China; Institute of Polymer Optoelectronic Materials and Devices, Guangdong Basic Research Center of Excellence for Energy and Information Polymer Materials, State Key Laboratory of Luminescent Materials and Devices, South China University of Technology, Guangzhou 510640, China; Dongguan Volt-Amp Optoelectronics Technology Co., Ltd., Dongguan 523000, China; Institute of Polymer Optoelectronic Materials and Devices, Guangdong Basic Research Center of Excellence for Energy and Information Polymer Materials, State Key Laboratory of Luminescent Materials and Devices, South China University of Technology, Guangzhou 510640, China; Institute of Polymer Optoelectronic Materials and Devices, Guangdong Basic Research Center of Excellence for Energy and Information Polymer Materials, State Key Laboratory of Luminescent Materials and Devices, South China University of Technology, Guangzhou 510640, China; Institute of Polymer Optoelectronic Materials and Devices, Guangdong Basic Research Center of Excellence for Energy and Information Polymer Materials, State Key Laboratory of Luminescent Materials and Devices, South China University of Technology, Guangzhou 510640, China; Dongguan Volt-Amp Optoelectronics Technology Co., Ltd., Dongguan 523000, China; Institute of Polymer Optoelectronic Materials and Devices, Guangdong Basic Research Center of Excellence for Energy and Information Polymer Materials, State Key Laboratory of Luminescent Materials and Devices, South China University of Technology, Guangzhou 510640, China

**Keywords:** non-doped, hot exciton, blue OLEDs, chrysene, reverse intersystem crossing

## Abstract

Host–guest doping is the mainstream technology for organic light-emitting diodes (OLEDs). Non-doped OLEDs, using a single material for both electron migration and exciton luminescence, promise simplified preparation, but lack efficient emitting-layer materials due to the concentration quenching of excitons (especially long-lifetime triplet excitons). This study compares two novel isomeric emitters (*p*TCN and *m*TCN) based on the hot-exciton mechanism. It shows that thermodynamically favorable excited-state alignments enable efficient high-lying reverse intersystem crossing (hRISC) from high-lying triplet states (*T_n_, n* ≥ 2) to singlet states (*S*_1_) with Δ*E_Tn_*_−_*_S_*_1_ > 0. The *p*TCN-based non-doped device exhibited an unprecedented maximum external quantum efficiency (EQE_max_) of 20.3% with CIE coordinates of (0.15, 0.07), while *m*TCN (unfavorable Δ*E_Tn_*_−_*_S_*_1_ < 0) only has 5.3% EQE_max_. Photophysical and excited-state dynamics studies confirm that the difference between the rates of the hRISC processes (∼1 × 10^8^ vs. 0.7 × 10^8^ s^–1^) gives rise to this performance gap.

## Teaser

Via the hot exciton mechanism, this study rapidly harnesses high-energy triplet excitons to realize ultra-high-efficiency non-doped blue OLEDs and simplify device fabrication processes.

## INTRODUCTION

Achieving high-efficiency blue emission continues to be the primary challenge in advancing organic light-emitting diodes (OLEDs)[1–3]. Typically, the blue emissive layer can be fabricated through co-evaporating host–dopant materials [4–8] or simply depositing a non-doped single-component emitter [9,10]. Compared with the conventional devices consisting of host and dopant in the emissive layer, a non-doped device can effectively eliminate the requirement for a precise host–dopant ratio control, mitigate energy losses during the energy-transfer process from host to dopant, avoid complex multistep processing that can enhance process reproducibility and so forth [11–13]. These combined merits have positioned the development of high-performance non-doped blue devices as the key research focus in OLED technology, driven by their potential to reconcile performance metrics with scalable production requirements. According to spin statistics theory [14], the singlet-to-triplet excitons are formed at a ratio of approximately 1:3 upon charge recombination in the emissive layer. During the electroluminescence (EL) process, the accumulation of excitons (particularly long-lived triplet excitons) typically results in triplet–triplet and/or triplet–singlet quenching, impeding the effective utilization of excitons. In order to achieve high-efficiency non-doped OLEDs, rapid triplet–singlet exciton conversion is crucial.

Effective strategies for utilizing dark-state triplet excitons include developing organometallic complexes and thermally activated delayed fluorescence (TADF) materials. The former rely on noble metals such as iridium and platinum, which leverage strong spin–orbit coupling (SOC) to enable phosphorescence emission at room temperature[15], while the latter utilize reverse intersystem crossing (RISC) under thermal activation conditions to achieve efficient fluorescence emission [16,17]. Despite these strategies having successfully enabled high-efficiency green and red emitters, the development of efficient and stable blue emitters remains elusive [18–21].

Recent advances in blue TADF emitters have demonstrated notable improvements in device stability [22–24]. In contrast, the hot-exciton mechanism offers a unique pathway for the efficient and rapid utilization of high-energy triplet excitons. In this mechanism, higher-lying triplet states (*T_n_, n* ≥ 2) are efficiently converted into singlet states (*S_m_, m* ≥ 1) through the high-lying RISC process, offering a conceptually advanced route toward stable and efficient blue emitters [25–28]. However, as the competition process of internal conversion (IC, from *T_n_* to *T*_1_) and intersystem crossing (ISC, from *S*_1_ to *T_n_*) cannot be exclusively avoided, the performances of the hot-exciton emitters reported so far are much lower than the theoretical 100% internal quantum efficiencies [29–37]. In this regard, it is highly appreciable to develop emitters not only with the characteristics of thermodynamically favorable downconversion with a positive *T_n_*–*S*_1_ energy gap (Δ*E_Tn_*_−_*_S_*_1_ > 0), but also associated with a large gap between the high-lying triplet states and the lowest triplet state (Δ*E_Tn_*_−_*_T_*_1_) to suppress the intramolecular IC process [38–40]. Based on the hot-exciton mechanism, a range of deep-blue emitters have been developed by using anthracene or pyrene as the core structure, which is connected with electron-donating or -withdrawing moieties at peripheral positions. Despite these deep-blue emitters exhibiting impressive external quantum efficiencies of ∼10% with Commission Internationale de l'Éclairage (CIE) 1931 chromaticity coordinate y (CIE_y_) < 0.10, the efficiencies are much lower than the requirement for state-of-the-art blue emitters [41–45].

**Figure 1.**
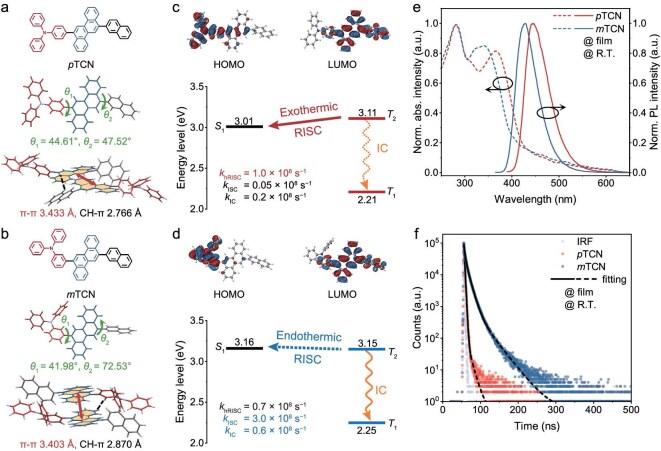
(a and b) Molecular structures and single-crystal structures of *p*TCN and *m*TCN. (c and d) Frontier orbital distributions and energy-level alignment of *p*TCN and *m*TCN. (e) Ultraviolet–visible (UV–vis) absorption and fluorescence spectra of *p*TCN and *m*TCN as thin films. (f) Transient PL decay spectra of *p*TCN and *m*TCN thin films at room temperature, along with the instrument response function.

Herein, we designed and synthesized two isomeric conjugated molecules based on a polycyclic aromatic hydrocarbon moiety of chrysene as the central unit. The four fused phenyl rings of the chrysene unit enable its extended π-conjugation system that confers exceptional fluorescence quantum yield and tunable optoelectronic characteristics [46–50]. The photophysical behaviors of the two isomers, namely *p*TCN and *m*TCN (molecular structures shown in Fig. 1a and b), can be strategically modulated by altering the *para*-position and *meta*-position of the peripheral triphenylamine moiety, respectively, as the electron-cloud distribution along the conjugated framework was closely related to their molecular geometry. Of particular interest is that the *p*TCN exhibited a higher *T_n_* than that of *S*_1_ (Δ*E_Tn_*_−_*_S_*_1_ > 0), enabling a more energetically favorable high-lying RISC (hRISC) process (*k*_hRISC_ = 1 × 10^8^ s^−1^) compared with its counterpart emitter, *m*TCN (Δ*E_Tn_*_−_*_S_*_1_ < 0, *k*_hRISC_ = 0.7 × 10^8^ s^−1^). Benefitting from the hybridized local and charge-transfer (HLCT) excited-state characteristics along with a high photoluminescent quantum efficiency (PLQY) of >80% for *p*TCN, the non-doped device based on *p*TCN exhibited a boosted maximum external quantum efficiency of 20.3% with CIE coordinates of (0.150, 0.073). To the best of our knowledge, this is the highest efficiency so far achieved for deep-blue non-doped OLEDs with CIE_y_ < 0.08, representing the great potential for its application.

## RESULTS AND DISCUSSION

The synthesis of the two isomeric emitters *p*TCN and *m*TCN was performed by using a two-step palladium-catalysed Suzuki coupling reaction in good overall yield (Scheme S1) and the molecular structures were confirmed by using nuclear magnetic resonance and mass spectroscopy (Figs S1–S10). Both emitters bear a conjugated molecular framework consisting of chrysene as the central unit with triphenylamine and naphthalene as the peripheral units. When compared with the reported chrysene-based derivative that is tethered with a strong electron-deficient benzonitrile moiety, the peripheral naphthalene moiety has a trivial effect on the electron-cloud distribution of both molecules [49]. The highest occupied molecular orbital (HOMO) and lowest unoccupied molecular orbital (LUMO) electron distributions of *p*TCN and *m*TCN are illustrated in Fig. 1c and d, respectively. The compound *m*TCN with *meta*-substituted triphenylamine presents a twisted geometry that weakens the conjugation and reduces the electron overlap of HOMO–LUMO. Additionally, solvatochromic analysis based on the Lippert–Mataga model yields excited-state dipole moments of 12.55 D for *p*TCN and 17.97 D for *m*TCN (Figs S11–S13 and Table S1), confirming the presence of a quasi-equivalent HLCT state that lends effective support to the hot-exciton mechanism [27,51].

The HOMO energy levels (*E*_HOMO_) of the two isomers were evaluated by using cyclic voltammetry measurements (Fig. S14 and Table S2), which were calculated to be −5.22 and −5.29 eV for *p*TCN and *m*TCN, respectively, according to the equation *E*_HOMO_ = −*e*(4.80 + *φ*_onset_ − *φ*_onset/Fc/Fc⁺_), where *φ*_onset_ and *φ*_onset/Fc/Fc⁺_ represent the oxidation and ferrocene/ferrocenium potential, respectively. The LUMO energy levels (*E*_LUMO_) are extrapolated by adding the optical band gap to the *E*_HOMO_, leading to *E*_LUMO_ values of −2.10 and −2.04 eV for *p*TCN and *m*TCN, respectively. The decomposition temperatures (*T*_d_, corresponding to 5% weight loss) were determined to be 448°C and 428°C for *p*TCN and *m*TCN, respectively, by using thermogravimetric analysis (Fig. S15). From the differential scanning calorimetry (DSC) characteristics, one can observe the glass-transition characteristics at 132°C and 120°C for *p*TCN and *m*TCN, respectively. No obvious melting and crystallization signals were observed in the DSC curves in multiple heating–cooling test cycles (Figs S16 and S17). These observations demonstrated that these two emitters had excellent thermostability, which was crucial for maintaining the quality and uniformity of the film during device operation.

As shown in the single-crystal structures (Fig. 1a and b, Figs S18 and S19 and Table S3), the steric hindrance from the peripheral triphenylamine groups, along with the inherently large molecular volume of the chrysene core, effectively prevents excessive π–π stacking of the fused rings, thereby mitigating fluorescence quenching. In both *p*TCN and *m*TCN, C–H···π interactions dominate the intermolecular interaction, with distances of 2.766 and 2.870 Å, respectively, while the π–π stacking distances are 3.442 Å for *p*TCN and 3.403 Å for *m*TCN. *p*TCN adopts a cross-stacked packing motif, featuring dihedral angles of 44.46° (*θ*_1_, between the meta-triphenylamine and chrysene) and 47.67° (*θ*_2_, between the naphthalene and chrysene). In contrast, *m*TCN exhibits a head-to-tail arrangement with dihedral angles of 41.98° (*θ*_1_) and 72.53° (*θ*_2_). Theoretical visualization of the weak intermolecular forces (Fig. S20) further confirms that van der Waals interactions dominate in both crystals, which is favorable for suppressing non-radiative decay pathways. As a result, both compounds exhibit relatively high PLQY in vacuum-deposited films (81.5% for *p*TCN and 51.9% for *m*TCN), which is slightly lower than in solution (90.1% for *p*TCN and 57.0% for *m*TCN). The observed photophysical differences between *p*TCN and *m*TCN are therefore likely attributable to their intrinsic excited-state characteristics.

As shown in Fig. 1e, both emitters showed deep-blue emission, while the photoluminescent (PL) peak of *p*TCN red-shifted to 444 nm compared with that of *m*TCN. The transient PL decay curve of *p*TCN demonstrated corresponding prompt and delayed fluorescence lifetimes of 1.8 and 10.1 ns, which were shorter than those of 2.6 and 64.6 ns for *m*TCN, respectively (Fig. 1f and Table 1). In addition, the time-resolved photoluminescence (TRPL) decay curves of the poly(methyl methacrylate) (PMMA)-doped films are nearly identical to those of the neat films, indicating that the excited-state dynamics are predominantly governed by the intrinsic molecular properties (Fig. S21). As a result, *p*TCN shows higher reversed intersystem conversion rates (*k*_hRISC_) of 1.0 × 10^8^ s^−1^ than that of 0.7 × 10^8^ s^−1^ for *m*TCN—much higher than the typical emitters based on the classical TADF mechanism (*k*_RISC_ ∼ 10^4^–10^6^ s^−1^) [4,6]. Moreover, the *p*TCN emitter presents an intersystem conversion rate (*k*_ISC_) of 0.5 × 10^7^ s^−1^ and an IC rate (*k*_IC_) from *T*_2_ to *T*_1_ of 0.2 × 10^8^ s^−1^, both of which are smaller than those of 3.0 × 10^8^ and 0.6 × 10^8^ s^−1^ for *m*TCN, respectively, indicating higher singlet-exciton-generation probability and suppressed triplet-exciton leakage.

**Table 1.** Photophysical properties of *p*TCN and *m*TCN.

Compound	*Φ*_PL_^a^ (%)	*Φ*_PF_^b^ (%)	*Φ*_DF_^b^ (%)	*τ*_PF_^c^ (ns)	*τ*_DF_^c^ (ns)	*k*_r_^b^ (× 10^8^ s^−1^)	*k*_nr_^b^ (× 10^8^ s^−1^)	*k*_ISC_^b^ (× 10^8^ s^−1^)	*k*_hRISC_^b^ (× 10^8^ s^−1^)	*k*_IC_^b^ (× 10^8^ s^−1^)
*p*TCN	81.5	80.8	0.7	1.8	10.1	4.6	1.0	0.05	1.0	0.2
*m*TCN	51.9	12.1	39.8	2.6	64.6	0.5	0.4	3.0	0.7	0.6

a Measured in a non-doped evaporated film of 40 nm. ^b^Calculated according to the methods described in Section S9. ^c^Obtained by fitting the TRPL decay curves using a double exponential decay function.

**Figure 2.**
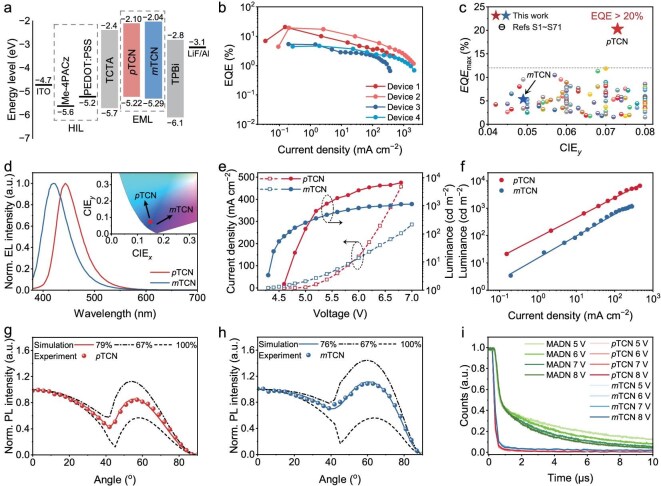
(a) Device-structure and energy-level diagrams of functional layers. (b) EQE versus current density characteristics. (c) EQE versus CIE_y_ plots of representative non-doped blue OLEDs. (d) Normalized EL spectra and corresponding CIE coordinate in operation. (e) Current density–voltage–luminance (*J*–*V*–*L*) characteristics. (f) Luminance as a function of the current density characteristics of devices. (g and h) Angle-dependent PL intensity for (g) *p*TCN and (h) *m*TCN. (i) Transient decay EL spectra.

Motivated by the rapid RISC process and high quantum yield (QY) of the films, the non-doped devices based on *p*TCN and *m*TCN were fabricated to evaluate their EL properties. The optimized structure was indium tin oxide (ITO)/Me-4PACz (5 nm) or poly(3,4-ethylenedioxythiophene): poly(styrenesulfonate) (PEDOT:PSS) (40 nm)/tris(4-carbazoyl-9-ylphenyl)amine (TCTA) (40 nm)/emitter (25 nm)/1,3,5-tri(phenyl-2-benzimidazolyl) benzene (TPBi) (30 nm)/LiF (1 nm)/Al (100 nm). Here, the ITO was used as an anode, a self-assembled layer of [4‐(3,6‐dimethoxy‐9*H*‐carbazol‐9‐yl)butyl]phosphonic acid (Me‐4PACz) or PEDOT:PSS was deposited on the top of the ITO layer and functioned as a hole-injecting layer, TCTA served as an electron-blocking and hole-transporting layer, TPBi acted as a hole-blocking and electron-transporting layer, LiF was utilized as an electron-injecting layer and Al was used as a cathode. An energy-level diagram and the EL performance are depicted in Fig. 2 and Figs S22–S27.

The non-doped device based on *m*TCN as the emissive layer exhibited a deep-blue emission that peaked at 422 nm and CIE coordinates of (0.159, 0.049), with a moderate maximum external quantum efficiency (EQE_max_) of 5.3%. In contrast, the *p*TCN device showed a remarkably high EQE_max_ of 20.3% with a slightly bathochromically shifted peak emission at 444 nm and CIE coordinates of (0.150, 0.073) (Fig. 2d, Fig. S22 and Table 2), and such a high device-efficiency value is reproducible (Fig. S23 and Table S4). As no observable low energy emission was realized in the EL spectra of ≤1000 nm, the potential formation of exciplex can be ruled out. Replacing the Me-4PACz with PEDOT:PSS also gives an impressively high EQE_max_ of 19.3% (Fig. 2b). We further verified the measurement reliability of the *p*TCN-based devices through Lambertian emission tests (Fig. S24). Nevertheless, to our knowledge, this is the highest EQE value so far achieved for deep-blue emission based on non-doped devices (Fig. 2c). Further study demonstrated that the *p*TCN-based device has better stability than the *m*TCN-based device (Fig. S25 and Table S5). When an acceleration factor of *n* = 1.75 is used, the estimated lifetime at 100 cd m⁻^2^ is 1030.5 h for the *p*TCN device, which is ∼12 times longer than that of the *m*TCN device. This improved device stability is likely attributed to the thermodynamically favorable hRISC process of *p*TCN, which can effectively suppress the accumulation of *T*_1_ excitons and reduce the probability of non-radiative quenching.

**Table 2.** Electroluminescent data of the optimized non-doped device based on *p*TCN and *m*TCN.

Device	HI layer	Emitter	*V*_on_^a^ (V)	*L*_max_ (cd m^–2^)	CE_max_^b^ (cd A^–1^)	PE_max_^c^ (lm W^–1^)	EQE_max/100/1000_^d^ (%)	CIE^e^ (*x, y*)	*λ*_EL_^f^ (nm)
1	Me-4PACz	*p*TCN	4.6	6644	13.8	9.0	20.3/9.9/4.7	(0.153, 0.073)	444
2	PEDOT:PSS	*p*TCN	5.2	1959	15.0	9.0	19.2/9.8/4.1	(0.147, 0.086)	448
3	Me-4PACz	*m*TCN	4.3	1156	1.7	1.3	5.3/2.8/1.3	(0.159, 0.049)	422
4	PEDOT:PSS	*m*TCN	7.4	771	1.9	0.8	4.6/2.4/–	(0.159, 0.067)	436

^a^Turn-on voltage at 1 cd m^−^^2^. ^b^Current efficiency. ^c^Power efficiency. ^d^External quantum efficiency at maximum, 100, and 1000 cd m^−2^, respectively. ^e^CIE coordinates taken at 1000 cd m^−2^. ^f^Peak wavelength of EL spectrum.

From the angle-dependent PL spectra shown in Fig. 2g and h, the out-coupling efficiency (*η*_out_) of the *p*TCN- and *m*TCN-based devices is calculated to be 31% and 21%, respectively [52], according to the equation of EQE_max_ = *γ* × *η_PL_* × *EUE* × *η*_out_ [53], where the photoluminescence quantum efficiency (*η_PL_*) represents the PL quantum efficiency and *EUE* represents the exciton utilization efficiency. With an optimized device architecture and the absence of any noticeable overshoot in the transient EL traces even under high current densities, the devices exhibit well-balanced carrier injection (Fig. S26) [54,55]. Therefore, it is reasonable to assume that the recombination efficiency (*γ*) in both devices is close to unity. The EUE values of *p*TCN and *m*TCN were calculated to be 80.2% and 50.0%, respectively. Transient electroluminescence measurements indicated that both the *p*TCN- and the *m*TCN-based devices exhibited a low delayed fluorescence proportion of ∼1%, which remained nearly unchanged upon enhancing the driving voltage from 5 to 8 V, associated with a relatively long delayed lifetime of 39 and 48 μs, indicating low triplet–triplet annihilation effects (Fig. S27). This observation is different from the classical triplet–triplet annihilation (TTA) emitter of 2-methyl-9,10-bis(naphthalen-2-yl)anthracene (MADN), which showed a much higher delayed fluorescence proportion of 43% and delayed lifetime of 25 μs (Fig. 2i). It is also noteworthy that both *p*TCN and *m*TCN exhibit a single-slope behavior in their double-logarithmic luminance–current density plots, with slopes of 0.720 and 0.778, respectively (Fig. 2f). This observation significantly deviates from the characteristic two-regime behavior in TTA-dominated emission processes, as TTA is intrinsically a bimolecular process, and up-conversion of the EL intensity typically exhibits two distinct regimes as a function of the current density: a quadratic dependence at low current densities, where mono-triplet decay predominates, and a linear dependence at high current densities, where bi-triplet interactions become dominant [56].

**Figure 3.**
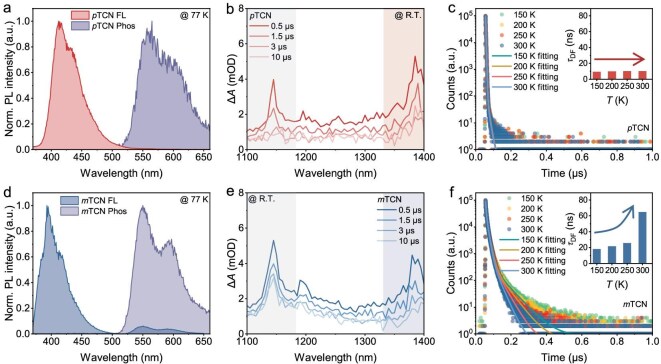
(a and d) Fluorescent (FL) and phosphorescent (Phos) spectra of *p*TCN and *m*TCN in toluene at 77 K. (b and e) Transient absorption spectra of the *p*TCN and *m*TCN solution at different time delays. (c and f) Transient PL decay spectra of *p*TCN and *m*TCN films measured at different temperatures. Inset: delayed fluorescence lifetimes of *p*TCN and *m*TCN at different temperatures.

An in-depth understanding of the EL-performance disparity between *p*TCN and *m*TCN necessitates the accurate determination of the exciton dynamic processes, which hinge on the nature of the excited states and the energy-level configuration. First, we calculated the energy-level alignments, natural transition orbitals and SOC values of the *S*_1_ and *T*_1_–*T*_5_ for both *p*TCN and *m*TCN (Figs S28 and S29). The results reveal that the two materials exhibit similar excited-state characteristics and SOC strengths. Given the significant discrepancy in their photophysical process rates, this finding demonstrates that energy-level alignment plays a more dominant role in governing the observed differences. Experimentally, the impact induced by energy-level alignment can be further validated. From the fluorescent and phosphorescent spectra of the two emitters in toluene at 77 K (Fig. 3a and d), the *S*_1_ and *T*_1_ energy levels were calculated to be 3.01 eV (412 nm) and 2.21 eV (560 nm) for *p*TCN, and 3.16 eV (393 nm) and 2.25 eV (550 nm) for *m*TCN, respectively. Although the *T*_1_ exciton lifetime reaches ∼1 second at 77 K, the large energy gap between *S*_1_ and *T*_1_ (Δ*E_T1_*_–_*_S_*_1_ > 0.8 eV) for both emitters effectively rules out the involvement of the TADF mechanism.

The nanosecond transient absorption (ns-TA) spectra of *p*TCN and *m*TCN in deoxygenated toluene were recorded upon excited at 355 nm (Fig. 3b and e, and Figs S30–S33). One can observe the relatively strong bands at 1380 and 1385 nm for *p*TCN and *m*TCN, respectively, upon delay for ≤10 μs. In combination with the measured *T*_1_ level and these signals, the high-lying *T*_2_ level was calculated to be 3.11 and 3.15 eV for *p*TCN and *m*TCN, respectively. Coinciding with the theoretical calculation, this energy-level arrangement was conducive to excitons undergoing the hRISC process from *T*_2_ to *S*_1_ for both *p*TCN and *m*TCN. Note that the *T*_2_ energy level was higher than the *S*_1_ value for *p*TCN, while the *T*_2_ energy level was lower than the *S*_1_ value for *m*TCN. This signified that the hRISC process of *p*TCN was thermodynamically favorable, as the *T*_2_ value of 3.11 eV is slightly higher than that of the *S*_1_ value of 3.01 eV. In contrast, the hRISC process of *m*TCN required additional energy to overcome the barrier, similarly to the TADF mechanism. The temperature-dependent transient PL decay spectra indicated that, as the temperature increased from 150 to 300 K, the delayed lifetime of the *p*TCN film remained nearly unchanged, while the delayed lifetime of *m*TCN increased from 18.2 to 64.6 ns (Fig. 3c and f, and Figs S34–S37). This was the result of the rate competition between the hRISC and ISC processes with opposite thermodynamic processes. For *p*TCN, the hRISC process is exothermic and therefore remains nearly unaffected as the temperature increases. In contrast, the hRISC process of *m*TCN exhibits an endothermic behavior, with *k*_hRISC_ increasing by ∼3-fold upon heating. The temperature-dependent photophysical parameters of *p*TCN and *m*TCN are summarized in Table S6.

**Figure 4.**
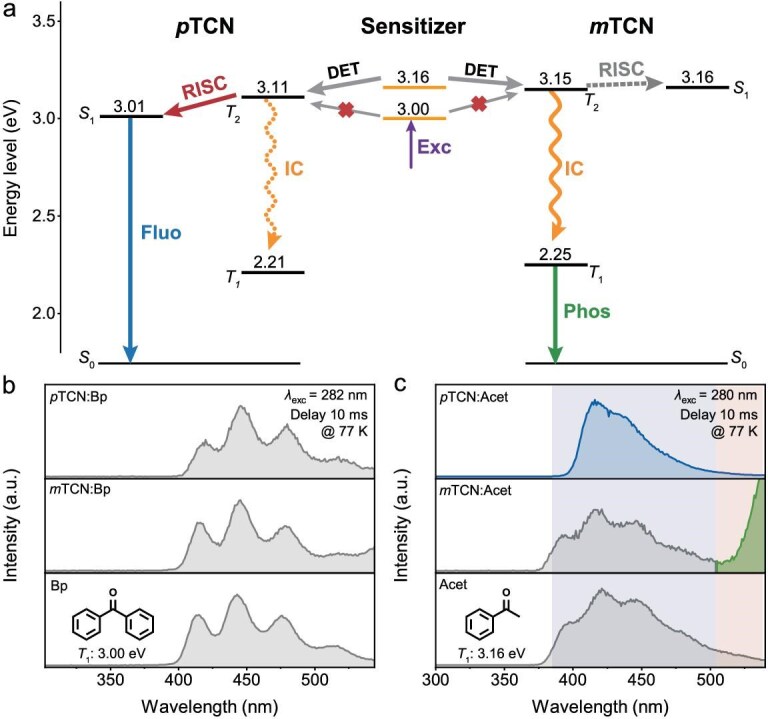
(a) Schematic diagram of ketone-sensitized triplet-exciton dynamic processes of *p*TCN and *m*TCN. (b) Top: delayed emission spectrum of the *p*TCN:Bp solution at 77 K; middle: delayed emission spectrum of the *m*TCN:Bp solution; bottom: phosphorescence spectrum of Bp. (c) Top: delayed emission spectrum of the *p*TCN:Acet solution at 77 K; middle: delayed emission spectrum of the *m*TCN:Acet solution; bottom: phosphorescence spectrum of Acet.

To investigate the kinetic behavior of triplet excitons in electroluminescence, we measured the steady-state spectra of these two emitters at a low temperature of 77 K by incorporating triplet sensitizers [57]. Herein, acetophenone (Acet, *T*_1_ = 3.16 eV) and benzophenone (Bp, *T*_1_ = 3.00 eV) were employed as the triplet sensitizer and the energy-level alignment is illustrated in Fig. 4a. For the Bp:*p*TCN or Bp:*m*TCN mixture solution, only emission corresponding to Bp was observed upon delay for 10 ms, indicating the lack of effective Dexter energy transfer from Bp to either *p*TCN or *m*TCN owing to the lower triplet energy of Bp (Fig. 4b). For the Acet:*p*TCN mixture solution, one observed a broad emission that can be exclusively attributed to the delayed fluorescence of *p*TCN, indicating that the high-lying triplet excitons of *p*TCN can effectively transfer to *S*_1_ through the hRISC process (Fig. 4c). In contrast, for the Acet:*m*TCN mixture solution, one can only observe the emission from the Acet sensitizer, while the fluorescent emission of *m*TCN was missed, demonstrating the lack of a pronounced hRISC process at a low temperature of 77 K. These findings agreed well with the thermodynamically unfavorable hRISC process for *m*TCN owing to its slightly lower *T*_2_ than *S*_1_. Considering that the *k*_hRISC_ and *k*_IC_ of *m*TCN are quite comparable, at a low temperature of 77 K, the competitive process of the IC process has priority over the hRISC process. Hence, it is rational to attribute the long-lifetime emission signal to *T*_1_ emission (phosphorescence) that is derived from the IC process (*T*_2_ → *T*_1_). Note that this signal did not appear at room temperature due to the accelerated non-radiative transition. Combining the triplet excitons kinetic processes of *p*TCN and *m*TCN, it can be concluded that only when the *T*_2_ energy level is higher than *S*_1_ can the fast triplet–singlet high-lying reversed intersystem conversion be achieved, which efficiently suppresses the IC process. These findings corroborated the hot-exciton process that enabled a maximum *EQE* of 20.3% with excellent blue color purity.

## CONCLUSION

In summary, by using chrysene as a core building unit, two novel hot-exciton materials *p*TCN and *m*TCN were designed and synthesized, with triphenylamine as the donor and naphthalene as the acceptor. Benefiting from the satisfactory aggregation state and HLCT excited-state characteristics, both chrysene derivatives exhibited high PLQY in the film with deep-blue emission. The experimental results revealed that there were different energy-level arrangements between the high-energy triplet and singlet states of *p*TCN and *m*TCN due to the different substitution sites of triphenylamine. The spontaneous exothermic hRISC process of *p*TCN, based on the slightly higher *T_n_* than *S*_1_ (Δ*E_Tn_*_−_*_S_*_1_ > 0), reached 1.0 × 10^8^ s^−1^, which could effectively inhibit the IC process from *T_n_* to *T*_1_. By contrast, for *m*TCN, as the *T_n_* state was lower than *S*_1_ (Δ*E_Tn_*_−_*_S_*_1_ < 0), the IC process was highly competitive compared with the hRISC process (*k*_hRISC_ = 0.7 × 10^8^ s^−1^), hindering the conversion of triplet excitons into singlet excitons in the EL process. As a result, the non-doped devices based on *p*TCN obtained a record EQE_max_ of 20.3% with CIE coordinates of (0.153, 0.073), which was much higher than that of *m*TCN at 5.3%. Our research emphasized the importance of spontaneous fast triplet–singlet excitons conversion in EL processes and reaffirmed that the hot-exciton mechanism is an effective strategy to achieve high-performance non-doped blue OLEDs.

## METHODS

The detailed preparation and characterization methods of the materials are available as Supplementary data.

## Supplementary Material

nwag056_Supplemental_File
