# Supplementary material for: Non-doped hot-exciton blue organic light-emitting diodes with efficiency over 20%
Source: Natl Sci Rev. 2026 Jan 31;13(6):nwag056. doi: 10.1093/nsr/nwag056 (PMC12994472; doi:10.1093/nsr/nwag056)
Supplement: nwag056_Supplemental_File [file nwag056_supplemental_file.pdf]

Supplementary Information

Non-doped hot exciton blue organic light-emitting diodes with efficiency over 20%

Mingke Li[1,†], Yulong Li[1,†], Yue Yu[1,2,*], Yichao Chen[1], Jianhui Pan[1], Feng Peng[2], Dezhi Yang[1], Dongge Ma[1], Lei Ying[1,2,*] and Yuguang Ma[1,*]

[1]Institute of Polymer Optoelectronic Materials and Devices, Guangdong Basic Research Center of Excellence for Energy and Information Polymer Materials, State Key Laboratory of Luminescent Materials and Devices, South China University of Technology, Guangzhou 510640, China;

[2]Dongguan Volt-Amp Optoelectronics Technology Co., Ltd., Dongguan 523000, China

***Corresponding authors**. E-mails: yuyue924@scut.edu.cn; msleiying@scut.edu.cn; ygma@scut.edu.cn

†Equally contributed to this work.

Table of Contents

Experimental Section

S1 – Synthesis

S2 – Solvatochromic Effects

S3 – Electrochemical Properties

S4 – Thermal Analysis

S5 – X-Ray Crystallography

S6 – Electroluminescence

S7 – Theoretical Calculations

S8 – Transient Absorption Spectroscopy

S9 – Photophysical Equations and Photoluminescence

References

Experimental Section

NMR and mass spectra

^{1}H NMR and ^{13}C NMR spectra were recorded on a Bruker AVANCE 500 spectrometer at 400 /500MHz and 101 MHz, respectively, using tetramethylsilane (TMS) as the internal standard and CD_2Cl_2 or $CDCl_3$ as the solvent. The matrix-assisted laser desorption ionization time-of-flight (MALDI-TOF) mass spectrum was measured using an AXIMA-CFR^{TM} plus instrument.

X-ray crystallography

X-ray data were collected on a Bruker APEX-II CCD diffractometer, The crystal was kept at 150.00 K during data collection. Using Olex2[1], the structure was solved with the SHELXT[2] structure solution program using Intrinsic Phasing and refined with the SHELXL[3] refinement package using Least Squares minimization. Table S3 provides summary details of the data collection and structure/refinement parameters. The X-ray crystallographic coordinates for structures reported in this study have been deposited at the Cambridge Crystallographic Data Centre (CCDC) under deposition numbers *p*TCN (2393754) and *m*TCN (2393754). These data can be obtained free of charge from The Cambridge Crystallographic Data Centre via www.ccdc.cam.ac.uk/data_request/cif.

Computational details

The initial geometry of *p*TCN or *m*TCN was extracted from their single crystal structures and then further optimized. The geometry of the ground state (S_0) was optimized at the density functional theory (DFT) level using the B2PLYP-D3(BJ) hybrid functional and def2-SVP basis. The calculations described above were performed using the Gaussian 16 software package.[4] Computations were performed using (time-dependent) density functional theory as implemented at the DSD-PBEP86/def2-SVP level of theory in ORCA 5.0.[5–8] The spin-orbit coupling matrix elements (SOCMEs) were calculated using TD-DFT as implemented in the ORCA 5.0 software package at the PBE0/Def2-TZVP level. The natural transition orbitals (NTOs) were generated from the wave function of the molecule at the excitation state using the Multiwfn[9–10] and VMD[11] programs. For the analysis of weak intermolecular interactions and reduced density gradient (RDG) isosurfaces, molecular structures were extracted from the single-crystal unit cell without further optimization, and single-point energy calculations were performed at the B3LYP-D3(BJ)/Def2-SVP level.

Thermal properties

Thermal gravimetric analysis (TGA) was performed on a METTLER TOLEDO TGA 2 apparatus from 30 °C to 800 °C under dry nitrogen at a heating rate of 20 °C min^{-1} while flushing with dry nitrogen. Differential scanning calorimetry (DSC) analysis was carried out using a TRIOS DSC 2500 instrument from 30 °C to 400 °C with a heating and cooling rate of 10 °C min^{-1} under a dry nitrogen flow rate of 50 ml min^{-1}.

Electrochemical measurements

Cyclic voltammetry (CV) was performed on a CHI760D electrochemical workstation with a Glassy Carbon working electrode and a Pt wire counter electrode at a scan rate of 50 mV s^{-1} against an Ag/Ag^{+} (0.01 M of $AgNO_3$ in acetonitrile) reference electrode in a nitrogen-saturated anhydrous dichloromethane solution of 0.1 mol/L Bu_4NPF_6 as the electrolyte.

Photophysical measurements

Solution absorption spectra were measured using a Shimadzu UV-2600 instrument. Fluorescence and time-correlated single-photon-counting experiments were carried out on Edinburgh Instruments FLS 980.

For organic films for photophysics, quartz substrates (steady-state PL and absorption, PL quantum, efficiency transient PL, and TA) were prepared, and they were cleaned with acetone and isopropyl alcohol with sonification for 10 min before loading them into an evaporator. Then, 40-nm-thick films were formed on glass and quartz substrates, respectively. The thermal evaporating process was conducted in a vacuum chamber under $<1.6 \times 10^{-5}$ Pa. Steady-state PL spectra at room temperature and 77 K, as well as the delayed phosphorescence spectra at 10 ms, were measured by a Horiba Fluoromax-4 spectrofluorometer with a xenon arc lamp at λ_{Ex} = 360 nm under a deaerated environment. The transient photoluminescence (PL) decay curves were obtained by FluoTime 300 (PicoQuant GmbH) with a Picosecond Pulsed UV-LASTER (P-C-375B) as the excitation source. The solid-state PL quantum efficiencies (Φ_{PL}) were measured on a FluoTime 300 (PicoQuant GmbH) equipped with an integrating sphere. The time-resolved emission spectra measurements and the steady-state spectra were conducted using an FLS980, with the film temperature controlled by an Oxford temperature controller and a cryostat.

Nanosecond transient absorption spectra were measured using the TranSpec-FE spectrometer system from Flash Photonics, Hong Kong. The pump light source was an Opolette 355 LD nanosecond laser from OPOTEK, with a pulse width of 7 ns and an excitation wavelength of 355

nm. The probe light source was a laser-driven, high-stability white light source (EQ99X LDLS) with an output wavelength range of 190-1000 nm. The spectral detector had a detection range of 350-1400 nm, with a spectral resolution of 0.1 nm, and the time window for measurements ranged from 0 to 36 μs. A sealed, four-sided quartz cuvette was used to hold the samples, and oxygen was removed by bubbling nitrogen gas through the sample solution inside a nitrogen-filled glovebox. The solvent used was toluene, with the pure sample solution concentration at 10^{-5} M.

Organic light-emitting devices

The electroluminescent devices were fabricated using a vacuum deposition method. The deposition equipment used was the organic metal thin film deposition system ZHDS-400, from Beijing Technol Science Co., Ltd. The substrates for the light-emitting devices were glass coated with indium tin oxide (ITO) with a thickness of 135 nm and a sheet resistance of 15 Ω. Before device fabrication, the ITO glass was ultrasonically cleaned in acetone, isopropyl alcohol, detergent, deionized water, and ethanol multiple times, followed by drying. Before use, the ITO substrates were treated with O_2 plasma for 2 minutes. During device fabrication, the pressure in the deposition chamber was kept below 2×10^{-5} Pa. The deposition rate for the organic materials was maintained at 1-2 Å/s, while the deposition rates for lithium fluoride (LiF) and aluminum (Al) were 0.1 Å/s and 4-5 Å/s, respectively. The deposition rates and film thickness were monitored in real-time using a quartz crystal microbalance from Inficon, USA.

The current density-voltage-luminance (I–V–L) characteristics of the devices were measured using a system composed of a Keithley 2400 source meter and a C200 chromameter, with real-time control provided by an SQC–310 monitor. The electroluminescence (EL) spectra were obtained using a PR745 spectroradiometer. The external quantum efficiency (EQE) was calculated based on the luminance, current density, and EL spectra, assuming Lambertian light emission.

To determine the out-coupling efficiencies, we first measured the refractive indices (n, k) of the functional materials and the emitters using a Mueller-matrix ellipsometer (ME-L, Wuhan Eoptics Technology Co.) over a wavelength range of 300–1000 nm. The neat films were prepared by evaporating on crystalline silicon and the thickness of each material film was 30 nm, and the refractive indices of the emitting layers were determined to be 1.75 ± 0.1 for *p*TCN and 1.78 ± 0.1 for *m*TCN, respectively. For measurement of the orientation of emitting dipoles in emitters, a setup RSQX-02 made by the Changchun Ruoshui Technology Development Co., Ltd. was used. To obtain the actual out-coupling efficiencies (η_{out}), we evaluated the radiative decay of electric dipoles embedded in the multilayer optical microcavity using the classical power spectrum (CPS) method.[12] This simulation incorporates the measured (n, k), emitting dipole distribution, and

device stack geometry. Under this validated multilayer model, the η_{out} values were calculated to be 31% for the *p*TCN device and 21% for the *m*TCN device, respectively.

S1 – Synthesis

Scheme S1 discusses the synthesis routes. All the reagents and solvents were purchased from commercial sources and used as received without further purification. The final products were subjected to vacuum sublimation to further improve purity before photoluminescence (PL) and electroluminescence (EL) properties investigations.

6,12-Bis(4,4,5,5-tetramethyl-1,3,2-dioxaborolan-2-yl)chrysene (Ch2B): 6,12-dibromochrysene (10 g, 25.9 mmol), Bis(pinacolato)diboron (19.73 g, 77.7 mmol, 3.0 equiv), KOAc (12.71 g, 129.5 mmol, 5 equiv), Pd(dppf)Cl_2 (0.57 g, 0.78 mmol, 0.03 equiv) and 250 mL 1,4-Dioxane were placed in a 500 mL round-bottom flask. The mixture was heated at 110 °C under nitrogen for 12 hours. The mixture was washed three times with 100 ml water and extracted with dichloromethane. The organic solution was dried by Mg_2SO_4, and then the solvent was evaporated. The residue was purified by column chromatography eluting with petroleum ether-dichloromethane mixtures to give Ch2B as a white solid (Yield: 11.57 g, 93%).^{1}H NMR (400 MHz, Chloroform-*d*) δ 9.37 (s, 2H), 8.96 (s, 4H), 7.67 (s, 4H), 1.50 (s, 24H). MS (m/z): calcd for $C_{30}H_{34}B_2O_4$, 480.26; found, 480.25 [M^+].

4,4,5,5-Tetramethyl-2-(12-(naphthalen-2-yl)chrysen-6-yl)-1,3,2-dioxaborolane (NaChB): In a 500 mL round-bottom flask, 2-bromonaphthalene (3.0 g, 14.49 mmol), Ch2B (9.04 g, 18.83 mmol, 1.3 equiv), K_2CO_3 (10.01 g, 72.44 mmol, 5 equiv), and Pd(PPh_3)$_4$ (0.50 g, 0.43 mmol, 0.03 equiv) were dissolved in 60 mL toluene, 20 mL ethanol, and 20 mL deionized water. The mixture was heated at 70 °C under nitrogen for 10 hours. After completion, it was cooled to room temperature, and then extracted three times with saturated saline and dichloromethane. The organic phase was dried with $MgSO_4$, and the solvent was removed by rotary evaporation. The crude product was purified by column chromatography and recrystallized to yield NaChB as a white solid. (yielding 3.2 g, 45.98%). ^{1}H NMR (500 MHz, Chloroform-*d*) δ 9.43 (s, 1H), 9.07 (s, 1H), 8.97 (s, 1H), 8.80 (s, 1H), 8.75 (s, 1H), 8.12 (s, 1H), 8.03 (s, 4H), 7.76 (d, J = 8.3 Hz, 4H), 7.68 (s, 2H), 7.57 (s, 3H), 1.52 (s, 12H). MS (m/z): calcd for $C_{39}H_{26}BO_2$, 480.23; found, 480.20 [M^+].

4-(12-(Naphthalen-2-yl)chrysen-6-yl)-N,N-diphenylaniline (*p*TCN): In a 250 mL round-bottom flask, 4-Bromotriphenylamine (3.0 g, 9.25 mmol), NaChB (5.33 g, 11.1 mmol, 1.2 equiv), K_2CO_3 (6.39 g, 46.26 mmol, 5 equiv), and Pd(PPh_3)$_4$ (0.32 g, 0.28 mmol, 0.03 equiv) were dissolved in 60 mL toluene, 20 mL ethanol and 20 mL deionized water. The mixture was heated at 90 °C under nitrogen for 10 hours. After completion, it was cooled to room temperature, and then

extracted three times with saturated saline and dichloromethane. The organic phase was dried with $MgSO_4$, and the solvent was removed by rotary evaporation. The crude product was purified by column chromatography and recrystallized to yield *p*TCN as a white solid. (yielding 3.95 g, 71.41%). ^{1}H NMR (400 MHz, Methylene Chloride-d_2) δ 8.92 (s, 2H), 8.77 (d, J = 20.6 Hz, 2H), 8.15 (s, 2H), 8.06 (s, 4H), 7.72 (s, 3H), 7.59 (s, 6H), 7.34-7.25 (s, 10H), 7.09 (s, 2H). ^{13}C NMR (101 MHz, Methylene Chloride-d_2) δ 139.11, 138.80, 133.57, 132.74, 131.20, 131.08, 129.34, 128.91, 128.61, 128.07, 127.74, 127.67, 126.91, 126.63, 126.52, 126.45, 126.42, 126.18, 124.53, 123.45, 123.06, 122.51. MS (m/z): calcd for $C_{46}H_{31}N$, 597.25; found, 597.08 [M^+].

3-(12-(Naphthalen-2-yl)chrysen-6-yl)-N,N-diphenylaniline (*m*TCN): In a 250 mL round-bottom flask, 3-Bromotriphenylamine (3.0 g, 9.25 mmol), NaChB (5.33 g, 11.10 mmol, 1.2 equiv), K_2CO_3 (6.39 g, 46.26 mmol, 5 equiv), and $Pd(PPh_3)_4$ (0.32 g, 0.28 mmol, 0.03 equiv) were dissolved in 60 mL toluene, 20 mL ethanol and 20 mL deionized water. The mixture was heated at 90 °C under nitrogen for 10 hours. After completion, it was cooled to room temperature, and then extracted three times with saturated saline and dichloromethane. The organic phase was dried with $MgSO_4$, and the solvent was removed by rotary evaporation. The crude product was purified by column chromatography and recrystallized to yield *m*TCN as a white solid. (yielding 4.34 g, 78.47%). ^{1}H NMR (400 MHz, Methylene Chloride-d_2) δ 8.87 (dd, J = 13.7, 8.5 Hz, 2H), 8.77-8.71 (d, J = 23.5 Hz, 2H), 8.14 (d, J = 1.8 Hz, 1H), 8.09 – 7.94 (m, 5H), 7.74 (s, 3H), 7.59 (d, J = 2.8 Hz, 4H), 7.47 (t, J = 7.8 Hz, 1H), 7.38 – 7.17 (m, 11H), 7.04 (s, 2H). ^{13}C NMR (101 MHz, Methylene Chloride-d_2) δ 147.96, 147.82, 142.15, 139.23, 139.04, 138.75, 133.55, 132.74, 131.17, 130.94, 130.82, 129.29, 128.90, 128.58, 128.06, 127.73, 127.66, 127.25, 126.92, 126.79, 126.65, 126.61, 126.52, 126.42, 126.18, 125.65, 124.42, 124.36, 123.46, 123.40, 122.97, 122.70, 122.46, 121.95. MS (m/z): calcd for $C_{46}H_{31}N$, 597.25; found, 597.08 [M^+].

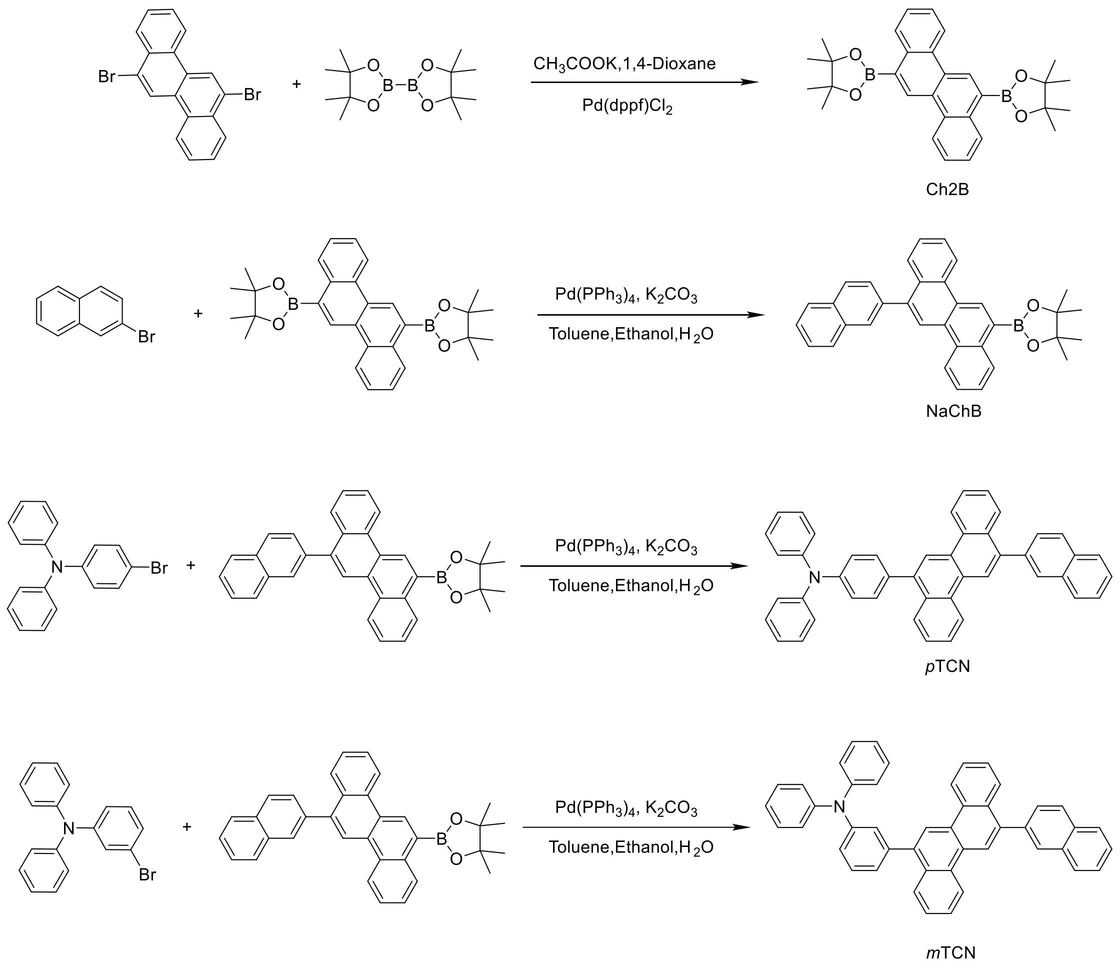

Scheme S1. Synthetic scheme of *p*TCN and *m*TCN.

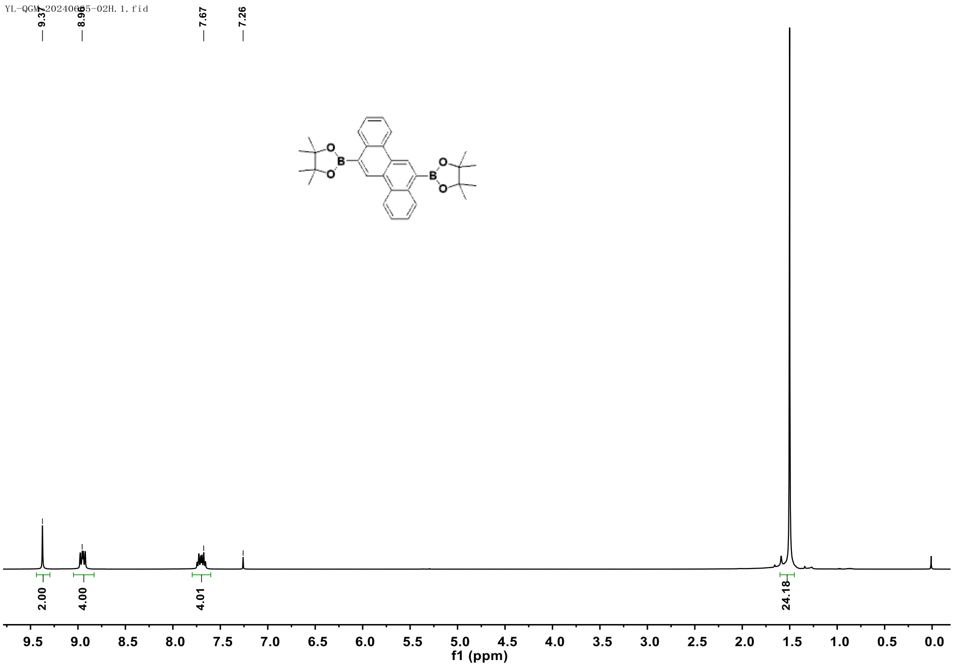

Figure S1. ^{1}H NMR spectra of Ch2B measured in deuterated $CDCl_3$.

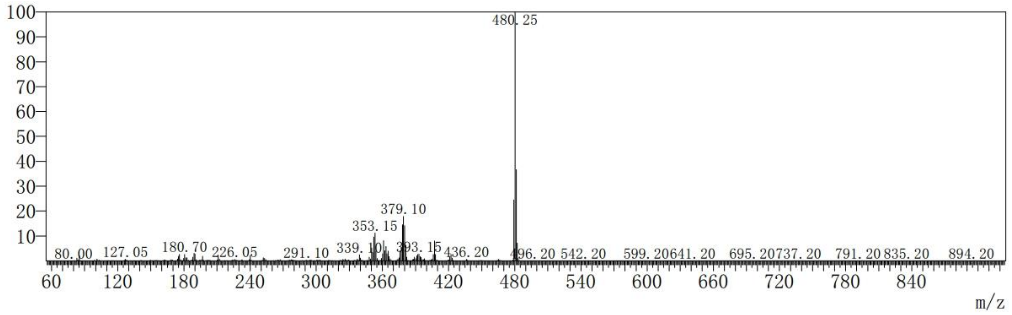

Figure S2. MALDI-TOF mass spectrum of Ch2B.

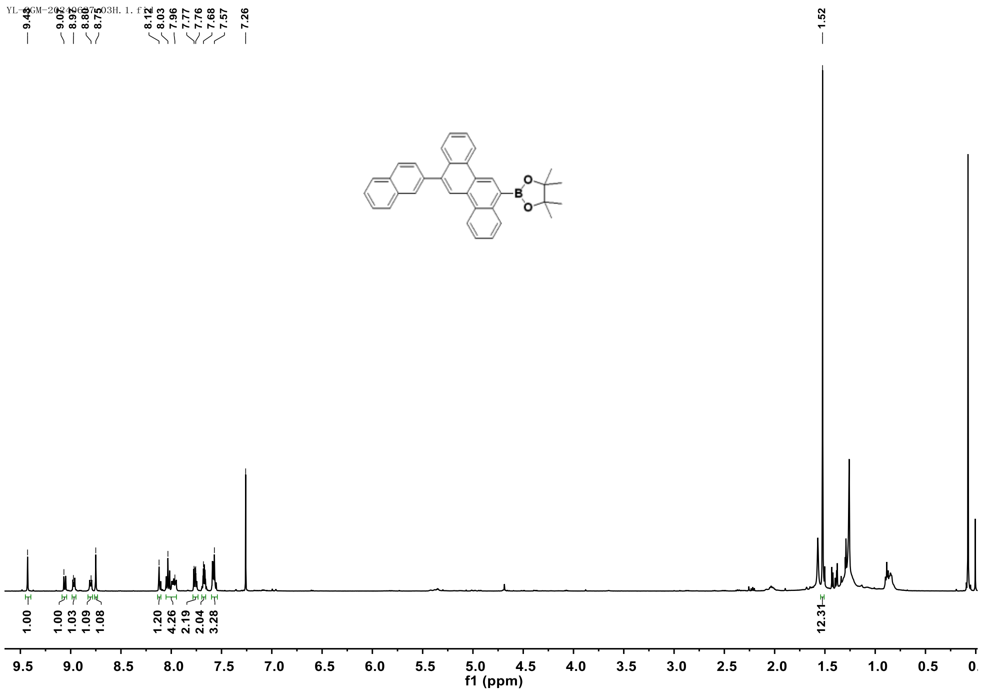

Figure S3. ^{1}H NMR spectra of NaChB measured in deuterated $CDCl_3$.

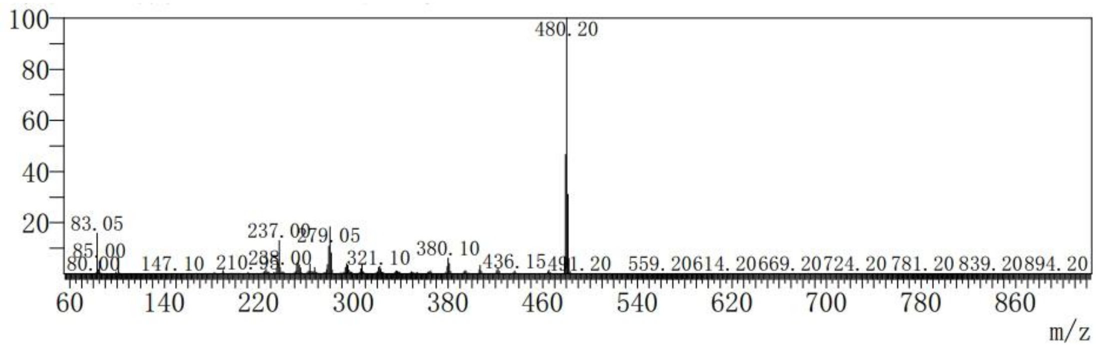

Figure S4. MALDI-TOF mass spectrum of NaChB.

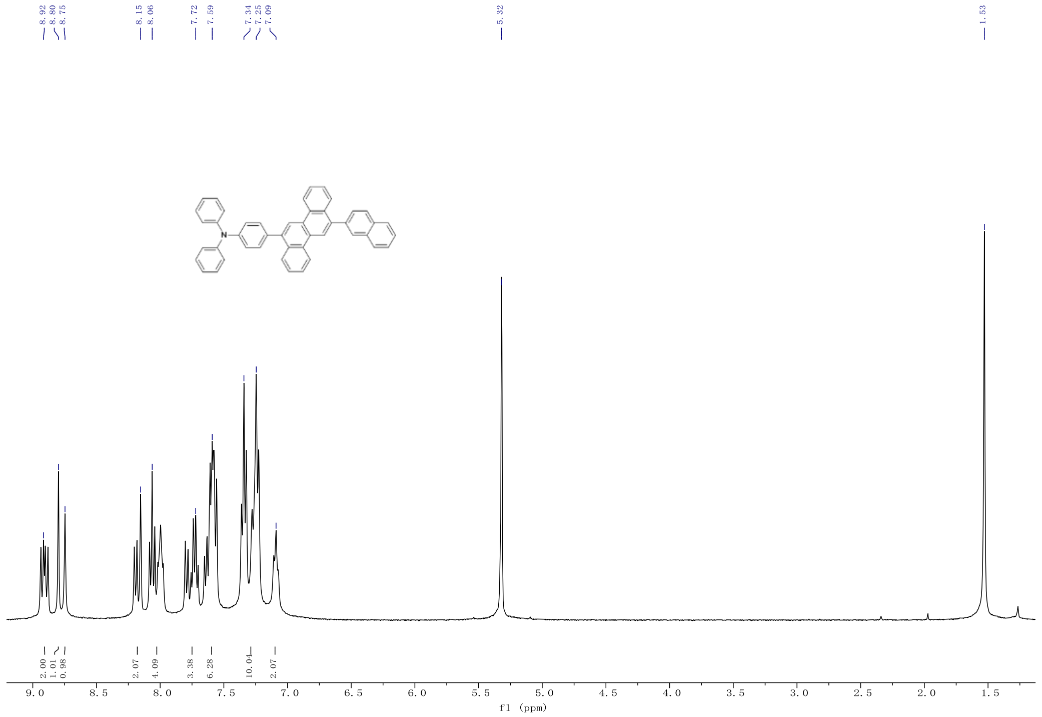

Figure S5. ^{1}H NMR spectra of *p*TCN measured in deuterated CD_2Cl_2.

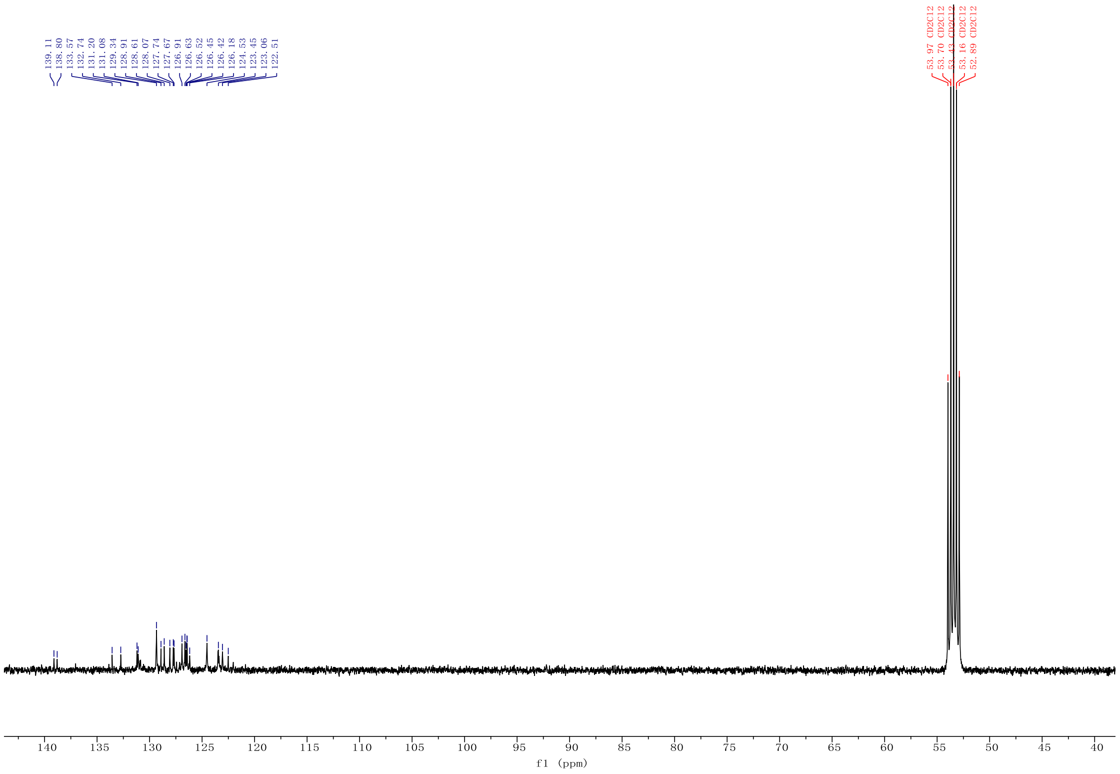

Figure S6. ^{13}C NMR spectra of *p*TCN measured in deuterated CD_2Cl_2.

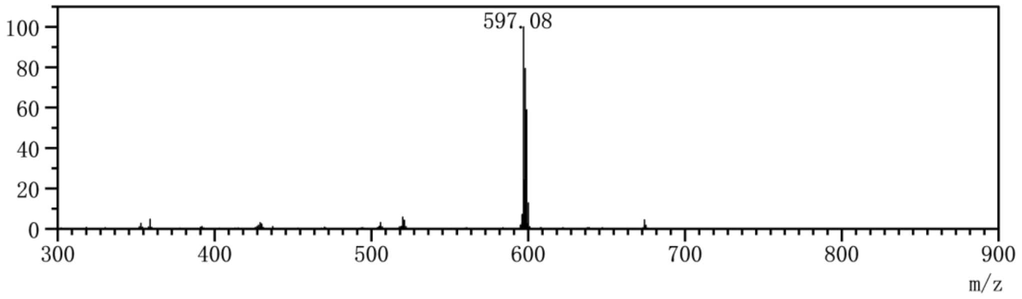

Figure S7. MALDI-TOF mass spectrum of *p*TCN.

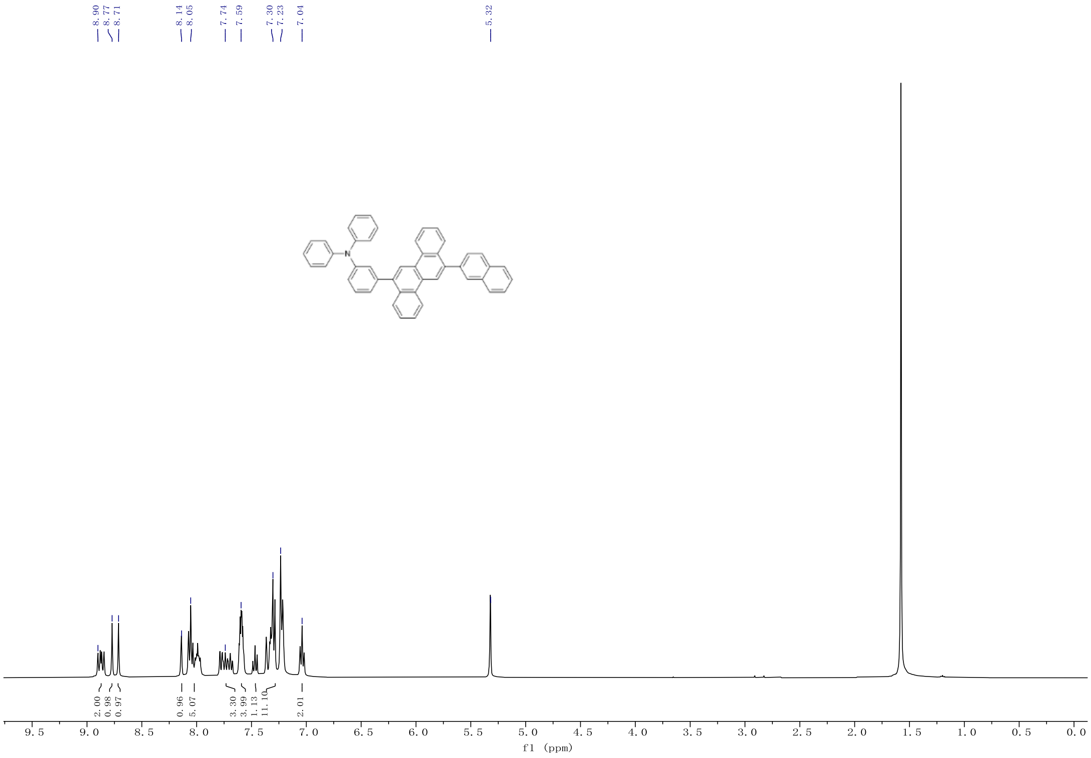

Figure S8. ^{1}H NMR spectra of *m*TCN measured in deuterated CD_2Cl_2.

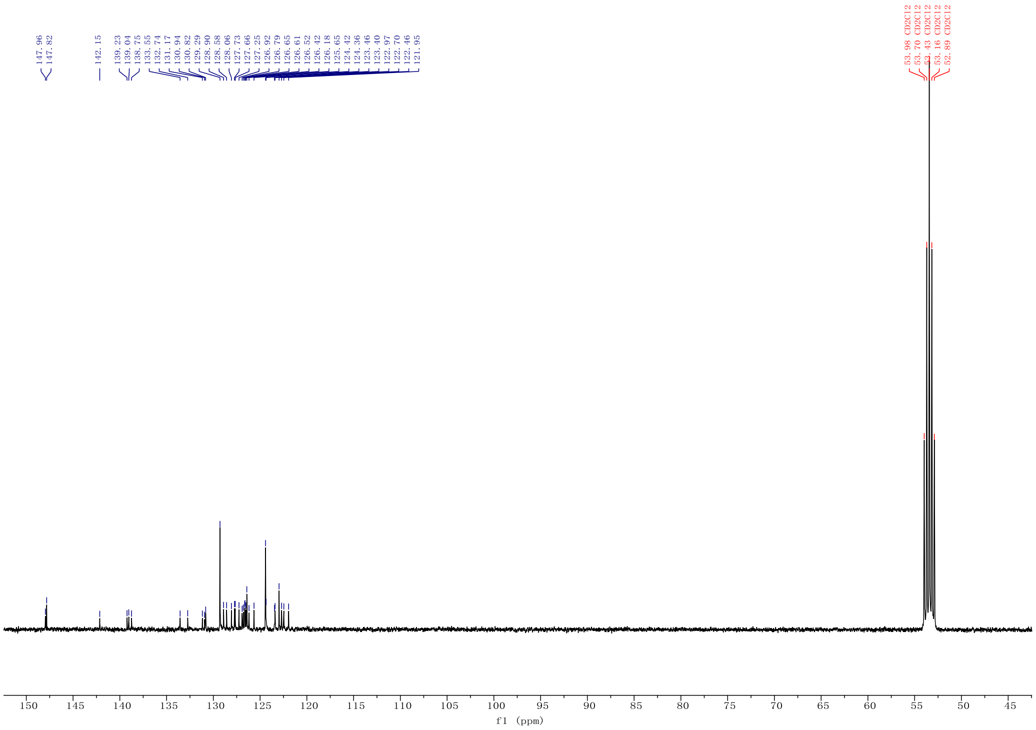

Figure S9. ^{13}C NMR spectra of *m*TCN measured in deuterated CD_2Cl_2.

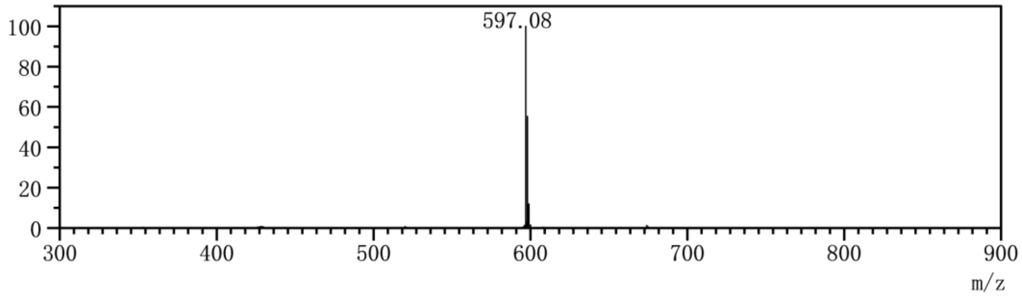

Figure S10. MALDI-TOF mass spectrum of *m*TCN.

S2 – Solvatochromic Effects

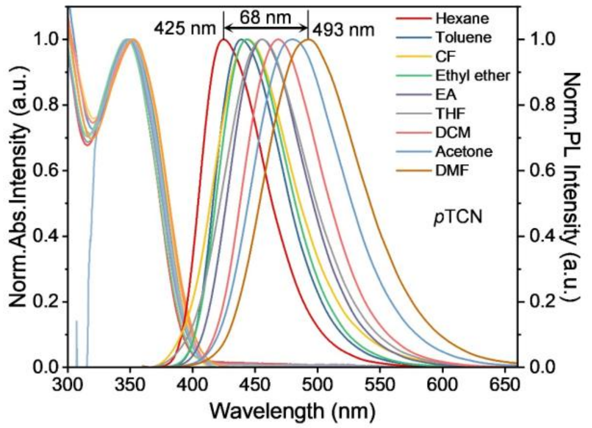

Figure S11. UV–Vis and PL spectra in different solvents (10^{-5} M) of *p*TCN.

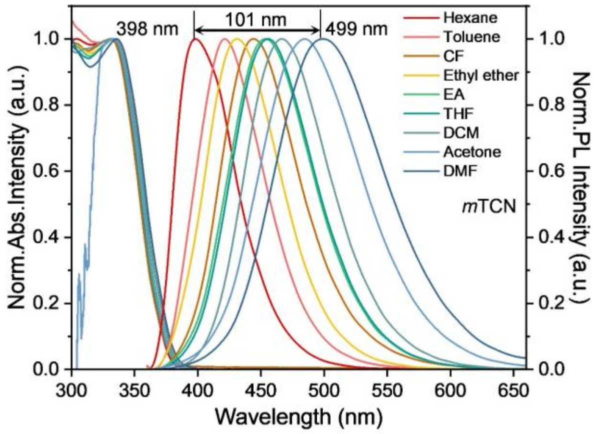

Figure S12. UV–Vis and PL spectra in different solvents (10^{-5} M) of *m*TCN.

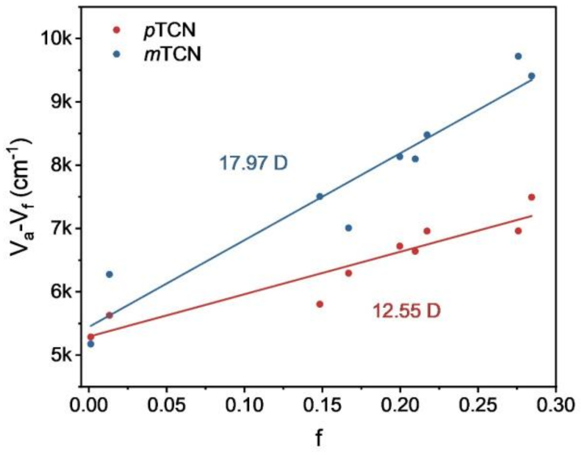

Figure S13. Solvatochromic Lippert-Mataga models of *p*TCN and *m*TCN.

The Lippert-Mataga model

The Lippert-Mataga model is estimated according to equation S1

$$hc(\nu_a - \nu_f) = hc(\nu_a^0 - \nu_f^0) + \frac{2(\mu_e - \mu_g)^2}{a_0^3} f(\varepsilon, n)$$

Equation S1

or

$$\mu_e = \mu_g + \left\{\frac{hca_0^3}{2} \cdot \left[\frac{d(\nu_a - \nu_f)}{2df(\varepsilon, n)}\right]\right\}^{\frac{1}{2}}$$

Equation S2

where μ_e is the dipole moment of excited state, μ_g is the dipole moment of ground state, h is the Plank constant, c is the light speed in vacuum, a_0 is the solvent Onsager cavity radius, $\nu_a - \nu_f$ is the Stokes shift, $f(\varepsilon, n)$ is the orientational polarizability of solvents and $f(\varepsilon, n) = [\frac{\varepsilon-1}{2\varepsilon+1} - \frac{n-1}{2n+1}]$. ε is the solvent dielectric constant and n is the solvent refractive index. μ_g was estimated by DFT of *p*TCN (μ_g = 0.48 D) and *m*TCN (μ_g = 0.22 D). The differential $\frac{d(\nu_a-\nu_f)}{2df(\varepsilon,n)}$ can be estimated based on the solvatochromic experiment data.

Table S1. Detailed absorption and emission peak positions of *p*TCN and *m*TCN in different solvents.

Solvents	ε	n	$f(\varepsilon,n)$	*p*TCN			*m*TCN		
				λ_a (nm)	λ_f (nm)	$\nu_a - \nu_f$ (cm^{-1})	λ_a (nm)	λ_f (nm)	$\nu_a - \nu_f$ (cm^{-1})
Hexane	1.9	1.375	0.0012	347	425	5289	330	398	5177
Toluene	2.37	1.496	0.014	352	439	5630	333	421	6277
Ethyl ether	4.34	1.352	0.167	347	444	6295	331	431	7009
Ethyl acetate	6.02	1.372	0.2	349	456	6723	331	453	8136
Tetrahydrofuran	7.58	1.407	0.21	350	456	6641	333	456	8100
Dichloromethane	8.93	1.424	0.217	353	468	6961	334	466	8480
Acetone	20.7	1.359	0.284	367	493	6963	336	499	9721
Acetonitrile	37.5	1.344	0.305	353	480	7495	333	485	9411

S3 – Electrochemical Properties

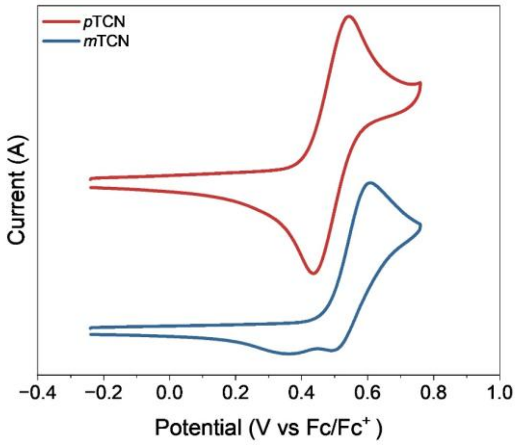

Figure S14. The cyclic voltammetry curves of *p*TCN and *m*TCN.

Cyclic voltammetry (CV) was performed on a CHI760D electrochemical workstation with a Glassy Carbon working electrode and a Pt wire counter electrode at a scan rate of 50 mV s^{-1} against a Ag/Ag^{+} (0.01 M of $AgNO_3$ in acetonitrile) reference electrode in a nitrogen-saturated anhydrous dichloromethane solution of 0.1 mol/L Bu_4NPF_6 as the electrolyte. The oxidation was determined to be 0.42 V and 0.49 V, respectively, against the ferrocenium/ferrocene (Fc/Fc^{+}) redox couple, corresponding to a HOMO level of −5.22 eV and −5.29 eV for *p*TCN and *m*TCN.

Table S2. Electrochemical data.

Compound	E^{ox}/V[a)]	HOMO/eV[b)]	LUMO/eV[c)]	E_g^{opt}/eV[d)]
*p*TCN	0.42	−5.22	−2.10	3.12
*m*TCN	0.49	−5.29	−2.04	3.25

[a)]Referenced to $E_{1/2}$ of the Fc/Fc^{+} redox couple; [b)]HOMO level calculated from CV potentials using the HOMO of ferrocene (–4.80 eV) a the standard, HOMO = −4.80 + ($-E^{ox}$); [c)]LUMO = HOMO + Eg^{opt}; [d)]Onset of the UV–Vis spectrum in toluene.

S4 – Thermalgravimetric analysis

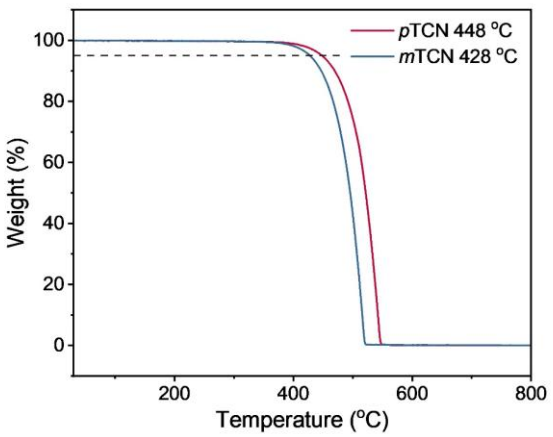

Figure S15. TGA of *p*TCN and *m*TCN.

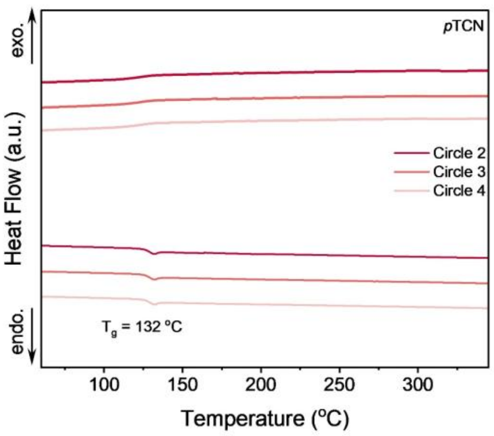

Figure S16. DSC of *p*TCN.

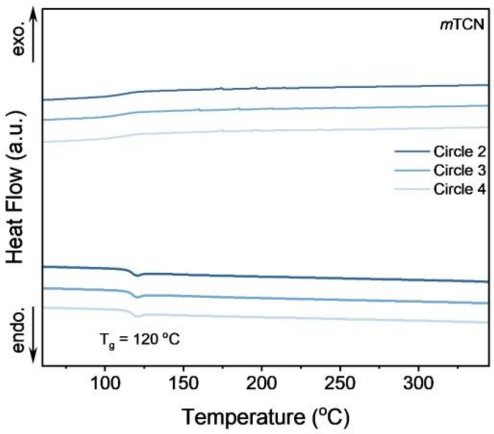

Figure S17. DSC of *m*TCN.

S5 – X-Ray Crystallography

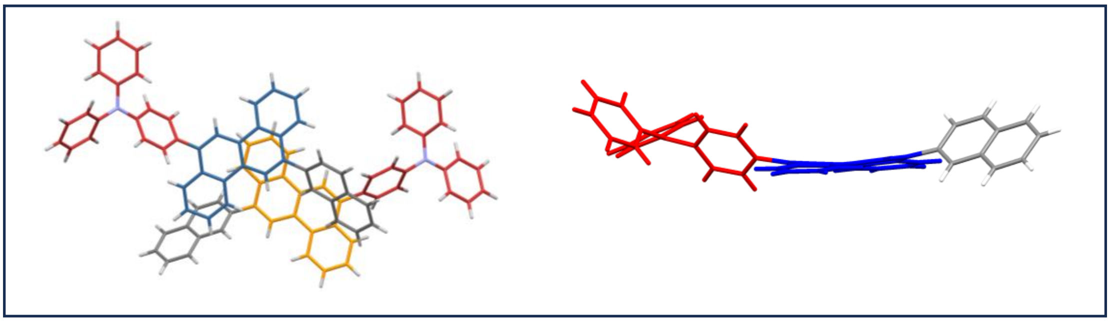

Figure S18. Crystal structure and Molecule packing of *p*TCN.

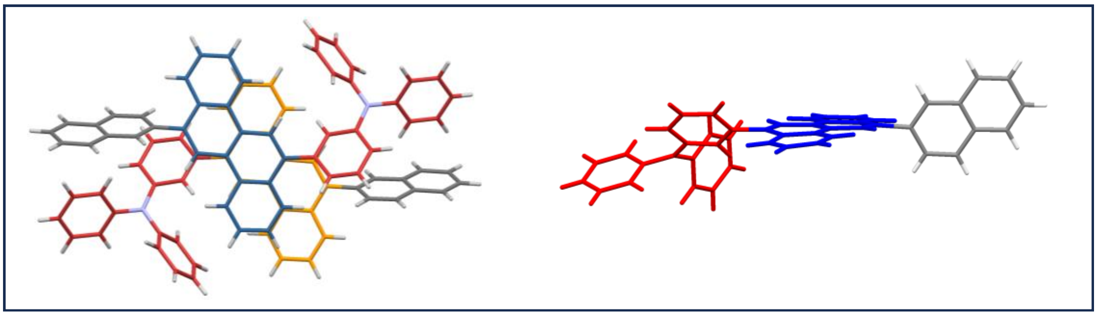

Figure S19. Crystal structure and Molecule packing of *m*TCN.

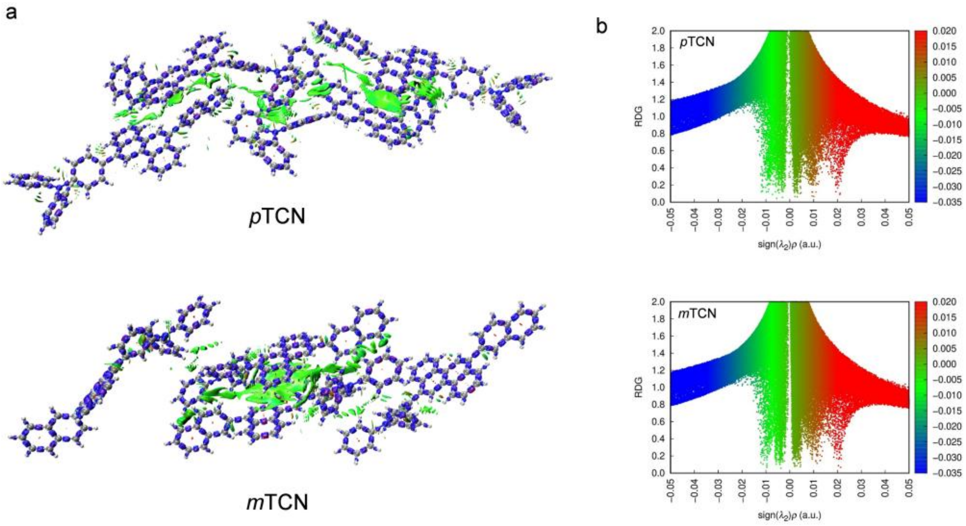

Figure S20. a) The isosurface representation of weak interactions in the single-crystal structure of *p*TCN (top) and *m*TCN (down). b) The RDG isosurface plot of *p*TCN (top) and *m*TCN (down) is based on the sign(λ_2)ρ function derived from its single-crystal structure. The molecular structure was directly extracted from the single-crystal unit cell without further optimization and subjected to single-point energy calculations in Gaussian. The resulting data were subsequently processed using VMD and Multiwfn.

Table S3. Crystal data and structure refinement of *p*TCN and *m*TCN

Parameter	*p*TCN	*m*TCN
deposition number	2393754	2393754
Empirical formula	$C_{46}H_{31}N$	$C_{46}H_{31}N$
Formula weight	597.72	597.72
Temperature/K	150.00	150.00
Crystal system	monoclinic	monoclinic
Space group	Pc	P21/c
a/Å	7.6269(11)	10.5676(8)
b/Å	39.801(8)	22.348(2)
c/Å	10.314(5)	13.7130(12)
α/°	90	90
β/°	91.011(16)	105.964(3)
γ/°	90	90
Volume/Å^3	3130.3(18)	3113.7(5)
Z	4	4
ρ_{calc}g/cm^3	1.268	1.275
μ/mm^{-1}	0.352	0.354
F(000)	1256.0	1256.0
Crystal size/mm^3	0.12 × 0.012 × 0.01	0.15 × 0.08 × 0.06
Radiation	GaKα (λ = 1.34138)	GaKα (λ = 1.34138)
2Θ range for data collection/°	3.862 to 118.708	6.772 to 118.85
Index ranges	$-9 \le h \le 9, -51 \le k \le 51, -13 \le l \le 13$	$-13 \le h \le 13, -28 \le k \le 28, -17 \le l \le 17$
Reflections collected	43252	38270
Independent reflections	13545 [R_{int} = 0.0579, R_{sigma} = 0.0540]	6843 [R_{int} = 0.0565, R_{sigma} = 0.0422]
Data/restraints/parameters	13545/2/848	6843/506/492
Goodness-of-fit on F^2	1.052	1.046
Final R indexes [I>=2σ (I)]	R_1 = 0.0436, wR_2 = 0.1041	R_1 = 0.0491, wR_2 = 0.1164
Final R indexes [all data]	R_1 = 0.0542, wR_2 = 0.1108	R_1 = 0.0683, wR_2 = 0.1281
Largest diff. peak/hole / e Å^{-3}	0.20/−0.19	0.31/−0.19

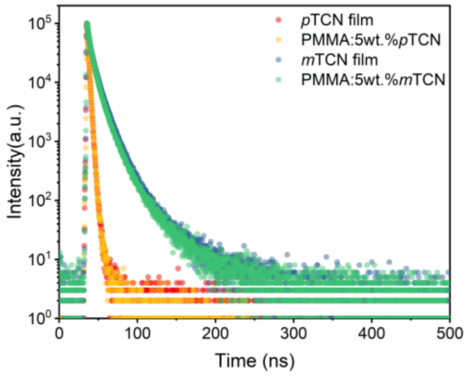

Figure S21: Transient photoluminescence (TRPL) decay curves of *p*TCN and *m*TCN in neat spin-coated films and in PMMA films doped with 5 wt.% emitter.

S6 – Electroluminescence

The optimized structure was ITO/Me-4PACz (40 nm)/TCTA (40 nm)/emitter (25 nm)/TPBi (30 nm)/LiF/Al. Indium tin oxide (ITO) was used as an anode, polymer polyethylene dioxythiophene:[4-(3,6-Dimethoxy-9H-carbazol-9--yl)butyl]phosphonic acid (Me-4PACz) functioned as a hole-injecting layer, tris (4-carbazoyl-9-ylphenyl) amine (TCTA) served as an electron-blocking and hole-transporting layer, 1,3,5-tri (phenyl-2-benzimidazolyl) benzene (TPBi) acted as a hole-blocking and electron-transporting layer, LiF was utilized as an electron-injecting layer, and Al was used as a cathode.

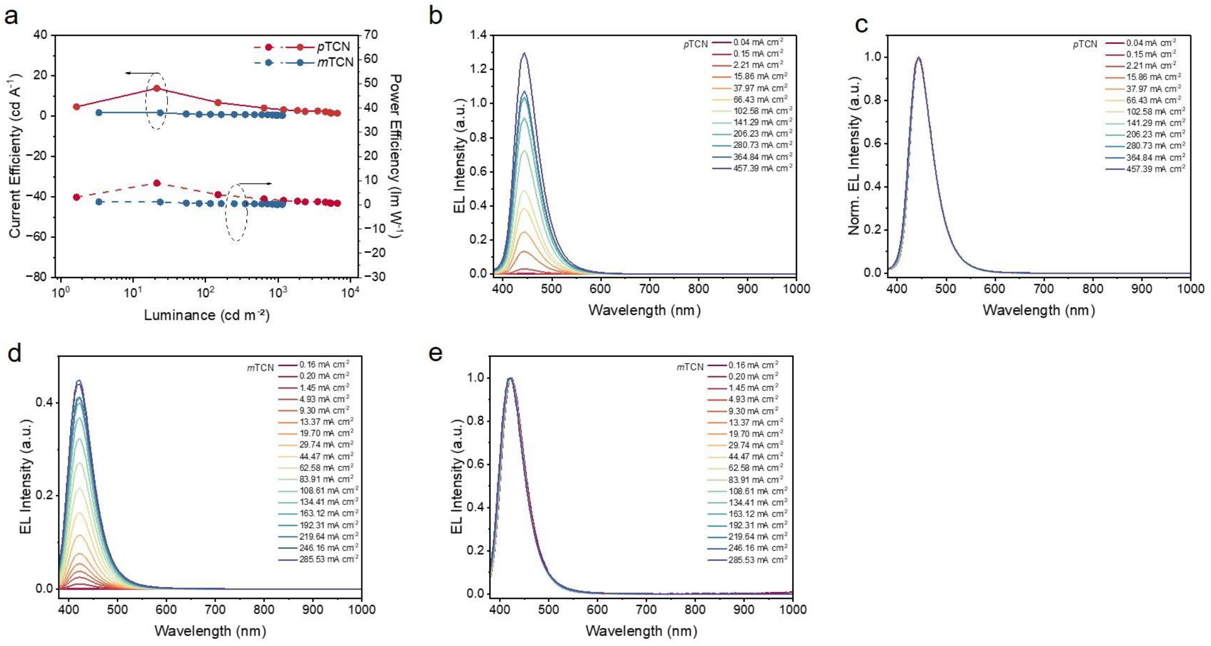

Figure S22. a). Current efficiency-luminance-power efficiency curves of *p*TCN and *m*TCN. b, d) The electroluminescence (EL) spectra of *p*TCN and *m*TCN, respectively, at different current densities. c, e) The normalized electroluminescence (EL) spectra of *p*TCN and *m*TCN, respectively, at different current densities.

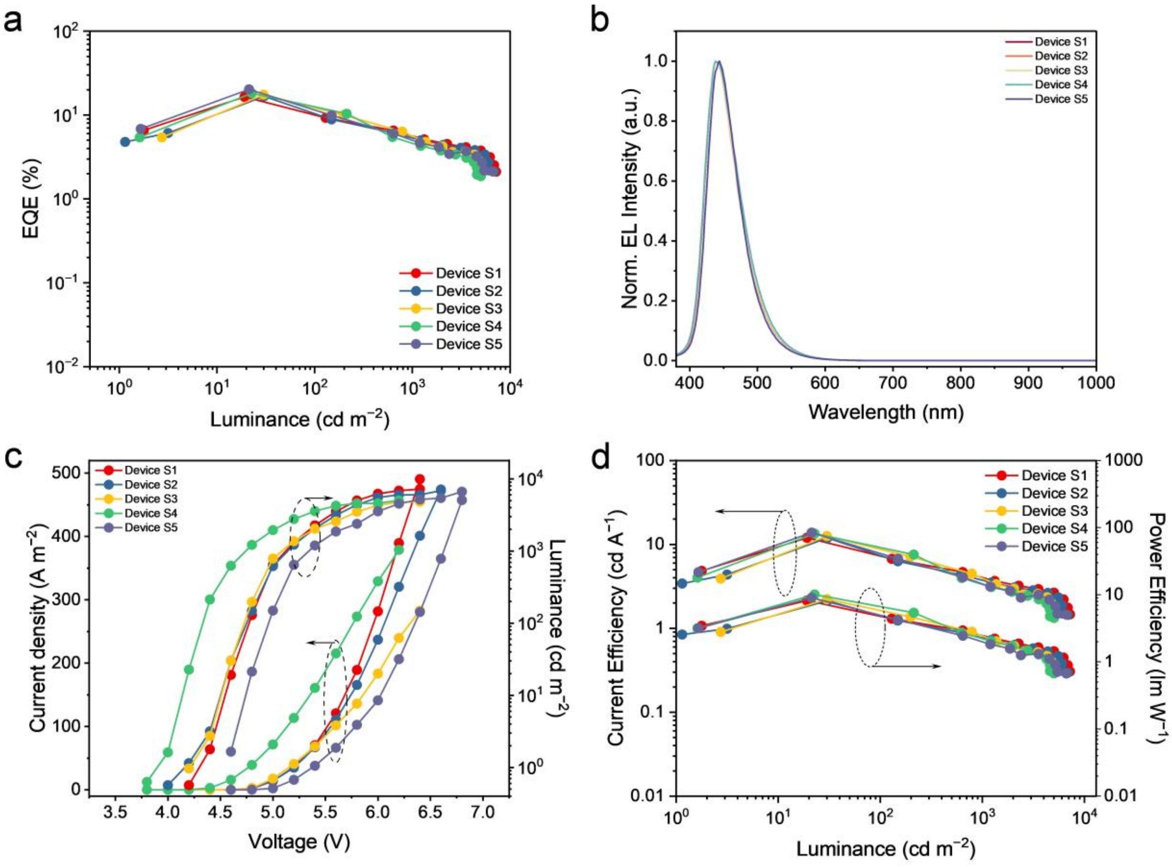

Figure S23. a) External quantum efficiency (EQE) and luminance curves of *p*TCN under different devices. b) Normalized electroluminescence (EL) curves of *p*TCN under different devices (at an initial luminance of 1,000 cd m^{-2}). c) Current density-voltage-luminance (J–V–L) characteristics of *p*TCN under different devices. d) Current efficiency-luminance-power efficiency curves of *p*TCN under different devices.

Table S4. Electroluminescent data of *p*TCN under different device.

Compound	V_{on}[a)] (V)	L_{max} (cd m^{-2})	CE_{max}[b)] (cd A^{-1})	PE_{max}[c)] (lm W^{-1})	$EQE_{max/100/1000}$[d)] (%)	CIE(x, y)[e)]	λ_{EL}[f)] (nm)	FWHM[g)] (nm)
Device S1	4.4	7202	12.0	8.2	16.5/9.3/5.1	(0.151, 0.080)	438	57
Device S2	4.2	6869	11.9	8.2	16.7/8.9/5.0	(0.151, 0.078)	438	56
Device S3	4.4	4826	12.6	8.6	17.5/10.0/4.8	(0.151, 0.080)	438	57
Device S4	4.0	5027	13.4	10.1	18.2/10.4/4.3	(0.151, 0.082)	438	57
Device S5	4.6	6644	13.8	9.0	20.3/9.9/4.7	(0.150, 0.073)	444	53

[a)] Turn-on voltage at 1 cd m^{-2}. [b)] Current efficiency. [c)] Power efficiency. [d)] External quantum efficiency at maximum, 100, and 1000 cd m^{-2}, respectively. [e)] CIE coordinates taken at 1,000 cd m^{-2}. [f)] Peak wavelength of EL spectrum. [g)] Full-width at half-maximum.

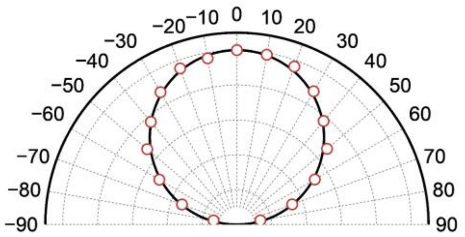

Figure S24. Angular distribution of the relative emission intensity of the device. The solid line represents the measured angular emission profile, while the dashed line corresponds to the ideal Lambertian distribution (cos θ). The device exhibits nearly Lambertian emission characteristics, indicating that the light intensity is strongest in the normal direction and gradually decreases with increasing angle, consistent with the behavior of a planar emitter. The device structure: ITO/PEDOT:PSS (40 nm)/TCTA (40 nm)/*p*TCN (25 nm)/TPBi (30 nm)/LiF/Al.

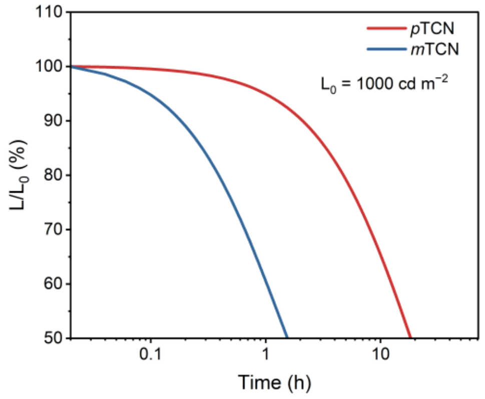

Figure S25. Lifetime curves of *p*TCN and *m*TCN devices.

Table S5. Electroluminescent data of the device based on the *p*TCN and *m*TCN.

Devices	LT_{50} (L_0 = 1000 cd m^{-2}) (h)	LT_{50} (L_0 = 100 cd m^{-2}) (h)
*p*TCN	18.3	1030.5
*m*TCN	1.5	86.7

To assess the operational stability of our emitters under continuous electrical driving, we measured the device lifetimes of both *p*TCN- and *m*TCN-based OLEDs. At an initial luminance of 1000 cd m^{-2}, the LT_{50} values (time to 50% of initial luminance) are 18.3 h for the *p*TCN device and 1.5 h for the *m*TCN device. Since practical display operation typically occurs near 100 cd m^{-2}, we further extrapolated the lifetimes using the commonly adopted luminance–lifetime acceleration

relation for blue OLEDs, T_{50}(100 cd m^{-2}) = T_{50} (1000 cd m^{-2}) × (1000/100)^n, with an acceleration factor of n=1.75. The extrapolated LT_{50} values at 100 cd m^{-2} are 1030.5 h for *p*TCN and 86.7 h for *m*TCN.

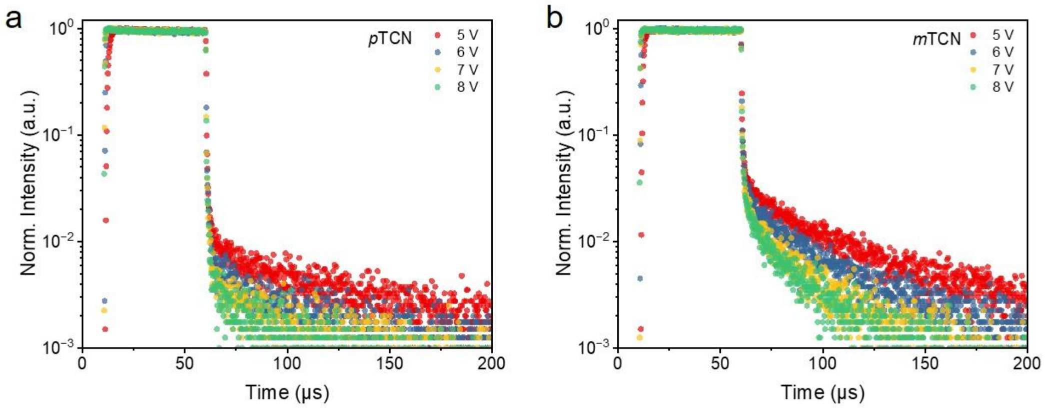

Figure S26. Transient electroluminescence (trEL) decays of the optimized devices based on (a) *p*TCN and (b) *m*TCN under different driving voltages. Device architecture: ITO / Me-4PACz (5 nm) / TCTA (40 nm) / emitter (25 nm) / TPBi (30 nm) / LiF / Al.

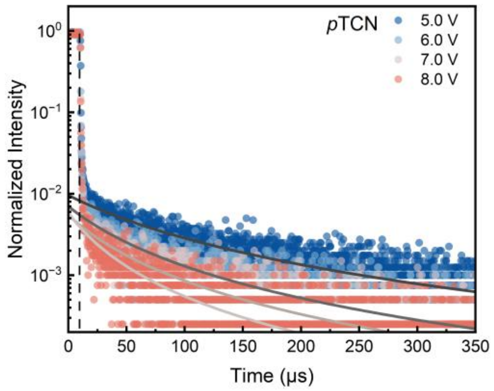

Figure S27. TREL decay curves of the *p*TCN device at different voltages; lines are the fits using the TTA model.

S7 – Theoretical Calculations

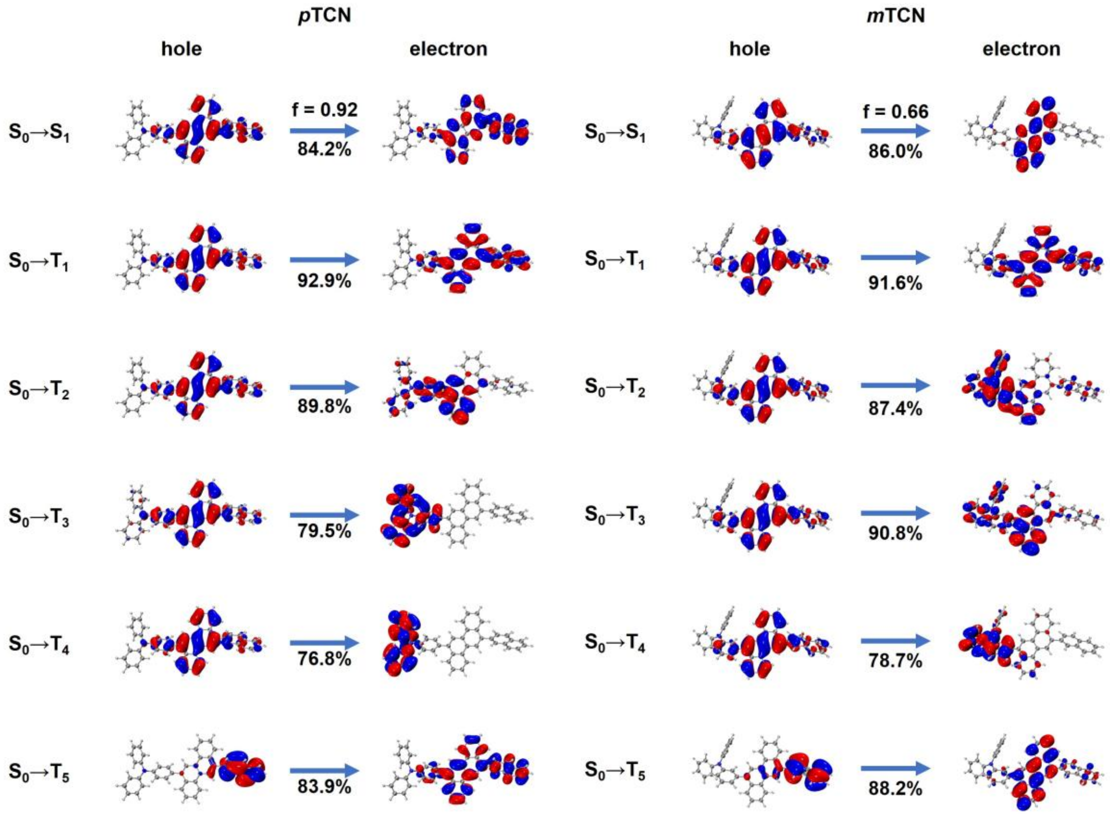

Figure S28. Natural transition orbital (NTO) representations of the hole and electron distributions for the $S_0 \rightarrow S_1$ and $S_0 \rightarrow T_n$ (n = 1–5) excitations of *p*TCN (left) and *m*TCN (right).

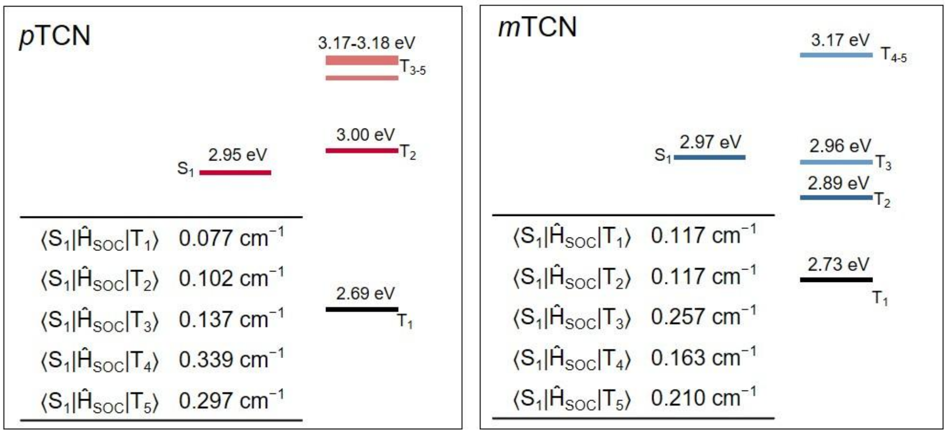

Figure S29. Energy-level diagrams and spin–orbit coupling (SOC) matrix elements between the S_1 state and the high-lying triplet states (T_1–T_5) for *p*TCN (left) and *m*TCN (right).

S8 – Transient Absorption Spectroscopy

In the 1100 ~ 1400 nm range, *p*TCN shows delayed absorption peaks around 4 microseconds at 1140 nm and 1380 nm, whereas *m*TCN exhibits absorption peaks at 1145 nm and 1385 nm that extend beyond the measurement range, preventing fitting. Analysis of these signals attributes the 1140 nm signal in *p*TCN to $T_1 \rightarrow T_3$ absorption and the 1380 nm signal to $T_1 \rightarrow T_2$ absorption. For *m*TCN, the 1145 nm signal is associated with $T_1 \rightarrow T_3$ absorption, and the 1385 nm signal corresponds to $T_1 \rightarrow T_2$ absorption peaks.

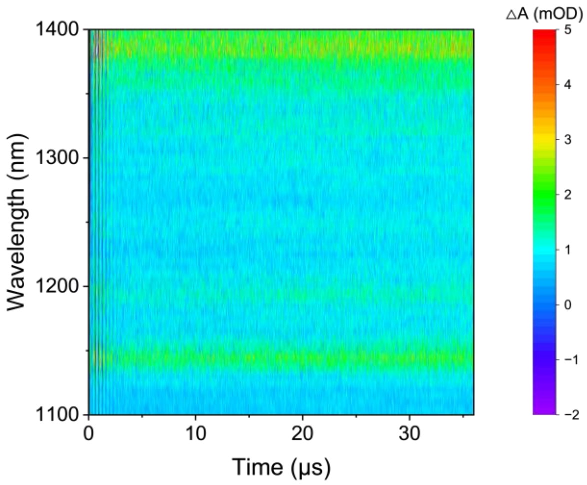

Figure S30. Nanosecond Transient absorption spectroscopy from 1100 nm to 1600 nm of *p*TCN (exc. 355 nm).

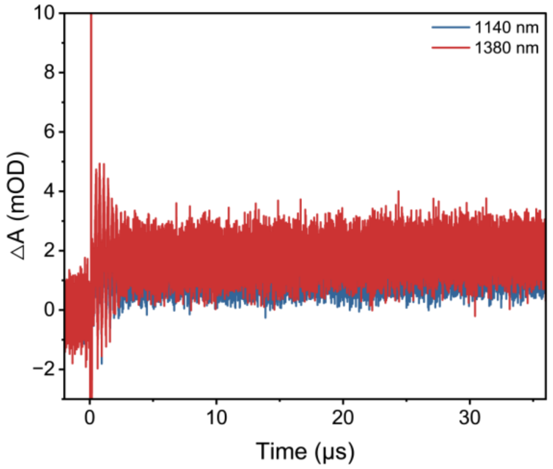

Figure S31. Triplet decay trace of *p*TCN (exc. 355 nm).

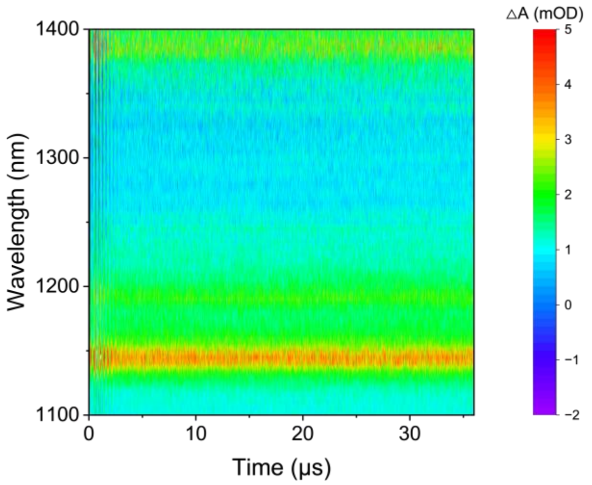

Figure S32. Nanosecond Transient absorption spectroscopy from 1100 nm to 1400 nm of *p*TCN (exc. 355 nm).

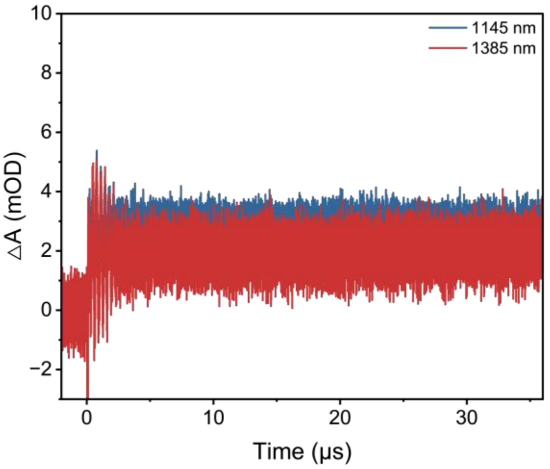

Figure S33. Triplet decay trace of *m*TCN (exc. 355 nm).

S9 – Photophysical Equations and Photoluminescence

The rate constants were determined using the following basic photophysical functions:

$\Phi_{PF} = \Phi_{PL} R_{PF}$

Equation S3

$\Phi_{DF} = \Phi_{PL} R_{DF}$

Equation S4

$k_r^S = \Phi_{PF}/\tau_{PF}$

Equation S5

$\Phi_{PL} = k_r^S/(k_r^S + k_{nr})$

Equation S6

$\Phi_{PF} = k_r^S/(k_r^S + k_{nr}^S + k_{ISC})$

Equation S7

$\Phi_{nr} = k_{nr}/(k_r^S + k_{nr}^S + k_{ISC})$

Equation S8

$\Phi_{ISC} = k_{isc}/(k_r^S + k_{nr}^S + k_{ISC})$

Equation S9

$\Phi_{hRISC} = \Phi_{DF}/\Phi_{ISC}$

Equation S10

$k_{hRISC} = \frac{k_p k_d \Phi_{DF}}{k_{ISC} \Phi_{PF}} = \Phi_{hRISC}/(k_{hRISC} + k_{IC}^{T_n})$

Equation S11

$k_p = 1/\tau_{PF}$

Equation S12

$k_d = 1/\tau_{DF}$

Equation S13

where Φ_{PL} is the photoluminescence quantum yield (PLQY); Φ_{PF} and Φ_{DF} are the prompt and delayed fluorescence efficiencies, respectively; τ_{PF} and τ_{DF} are the prompt and delayed fluorescent lifetimes, respectively, obtained by fitting the transient PL decay curves using a double exponential decay function; and k_r^S, k_{nr}^S, k_{ISC}, k_{hRISC}, and $k_{IC}^{T_n}$ are the rates of fluorescence decay, nonradiative processes, intersystem crossing, reverse intersystem crossing, and internal conversion process from T_2 to T_1, respectively. R_{prompt} and $R_{delayed}$ are the component ratios for prompt and delayed fluorescence, respectively. $R = A_1\tau_{PF}/(A_1\tau_{PF} + A_2\tau_{DF})$ and $R_{DF} = 1 - R_{PF}$, where A_1 and A_2 are the pre-exponential amplitudes for τ_{prompt} and τ_{prompt}, respectively.

Photoluminescence

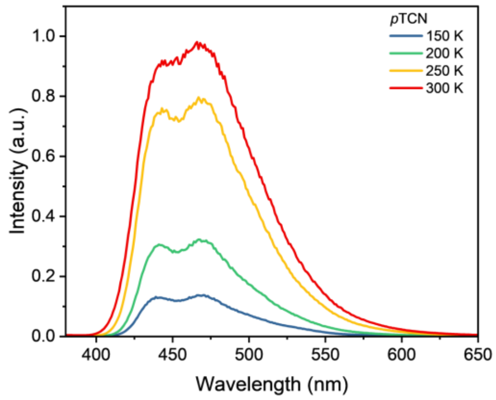

Figure S34. PL spectra in different temperatures from 150 K to 300 K of *p*TCN.

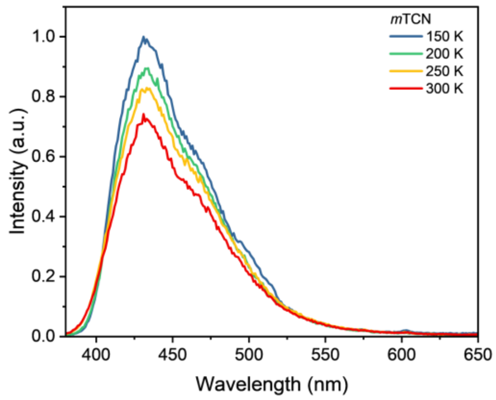

Figure S35. PL spectra in different temperatures from 150 K to 300 K of *m*TCN.

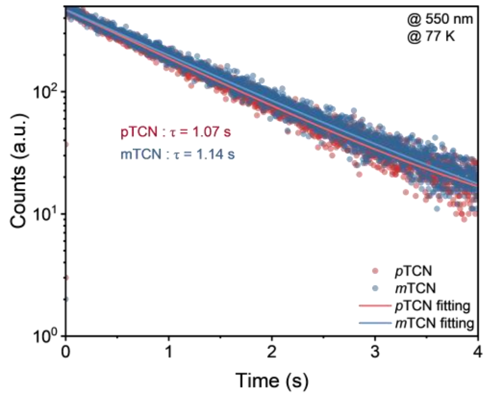

Figure S36: Transient PL decay spectra excited at 370 nm by a xenon lamp in toluene solution at 77 K, with a 10 ms delay and a 4 s time window (detected at 550 nm).

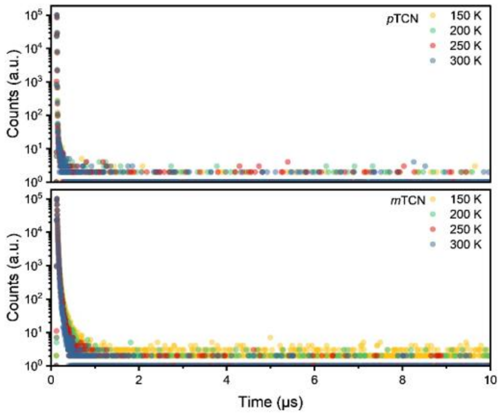

Figure S37: Transient emission spectra of *p*TCN (top) and *m*TCN (bottom) neat films at different temperatures, with a time window of 10 microseconds. (Exc.: 370 nm).

Table S6. The transient PL decay data of *p*TCN and *m*TCN films at different temperatures.

Compound	Temperature [K]	Φ_{PL}[a)] [%]	Φ_{PF}[b)] [%]	Φ_{DF}[b)] [%]	τ_{PF}[c)] [ns]	τ_{DF}[c)] [ns]	k_r[b)] [×10^8 s^{-1}]	k_{nr}[b)] [×10^8 s^{-1}]	k_{ISC}[b)] [×10^8 s^{-1}]	k_{RISC}[b)] [×10^8 s^{-1}]	k_{IC}[b)] [×10^8 s^{-1}]
*p*TCN	150	10.0	9.9	0.1	1.7	9.4	0.6	5.2	0.08	1.1	9.7
	200	23.9	23.6	0.2	1.8	9.9	1.3	4.3	0.06	1.0	3.2
	250	62.3	61.7	0.6	1.8	10.0	3.5	2.1	0.06	1.0	0.6
	300	81.5	80.8	0.7	1.8	10.1	4.6	1.0	0.05	1.0	0.2
*m*TCN	150	66.9	25.2	41.7	4.6	18.2	0.5	0.3	1.4	1.5	0.7
	200	60.5	20.5	40.1	3.9	21.5	0.5	0.3	1.7	1.4	0.9
	250	58.5	16.6	41.9	3.1	25.7	0.5	0.4	2.3	1.4	1.0
	300	51.9	12.1	39.8	2.6	64.6	0.5	0.4	3.0	0.7	0.6

[a)] Measured in a non-doped evaporated film of 40 nm. [b)] Calculated according to method described in S9**.** [c)] Obtained by fitting the transient PL decay curves using a double exponential decay function.

Table S7. Summary of highly efficient non-doped blue fluorescent OLEDs reported in recent years with EQE and CIEy ≤ 0.08.

Device	V_{on} (V)	L_{max} (cd A^{-1})	EQE_{max} (%)	EL (nm)	CIE_x	CIE_y	Ref.
*p*TCN	4.6	6644	20.3	444	0.15	0.07	This Work
*m*TCN	4.3	1156	5.3	422	0.16	0.05	This Work
PPITPh	3	18637	11.83	436	0.15	0.07	13
SP	3	13488	11.3	436	0.158	0.068	14
2M-ph-pCzAnBzt	3.4	3085	10.44	450	0.151	0.057	15
PHPYCZ	3.4	7917	9.5	418	0.154	0.049	16
FIP-CZ	3.3	9388	9.5	402	0.1703	0.0586	17
TPATPA-CNPPI	2.6	15892	8.96	457	0.16	0.07	18
2PPIAn	3	-	8.9	444	0.15	0.06	19
PIpPI	3	17560	8.84	428	0.15	0.07	20
PPIDPhC	3	4065	8.56	424	0.17	0.08	21
PPI-3-SBF	3	16365	8.41	428	0.16	0.07	22
PPI-PPIPCz	3.4	13820	8.1	-	0.15	0.07	23
2M-pCzAnBzt	3.4	2855	8.08	450	0.151	0.061	15
4PPIAn	2.9	-	8	444	0.149	0.068	19
PIPDMePBO	3.1	12212	8	427	0.156	0.048	24
ATDBF	2.61	-	7.93	439	0.15	0.06	25
PPIDPhO	3	4149	7.9	436	0.16	0.08	21
PPI-PPITPA	3.2	13950	7.7	-	0.15	0.08	23
BICZ	3	10780	7.67	422	0.153	0.063	16
DTPCZTZ	4	2514	7.6	424	0.17	0.06	26
MACN	-	3942	7.51	436	0.154	0.075	27
CN-TPB-TPA	3	4639	7.27	448	0.15	0.08	28
PPI-2TPA	3	14850	7.2	440	0.15	0.063	29
9-PCZCFOXZ	3.2	5740	7.2	417	0.1537	0.0757	30
CSiTPI	3.2	2445	7.1	404	0.16	0.06	31
ATPNF-1	2.9	-	7	446	0.15	0.07	32
C2MPI			6.97	404	0.161	0.063	33
TPIAnCN			6.84	438	0.15	0.07	34
TPA-PI-SBF	3.1	4901	6.76	448	0.152	0.059	35
FlCz			6.61		0.16	0.07	36
2FPPIDPA	2.8	15560	6.49	430	0.156	0.046	37
PPI-2NPA			6.33	448	0.151	0.066	29
PITPh	3.3	5634	6.1	424	0.16	0.06	13
pCzAnN	3.3	7750	6	-	0.15	0.07	38
CzB-MOPPI	4.8	-	5.97	435	0.16	0.08	39

TPBPPI-PBI	3.9	-	5.94	429	0.16	0.059	40
2Na-CzCN	3.7	4759	5.92	398	0.15	0.06	41
3-PCZOXZ	3.5	6156	5.9	417	0.155	0.0732	30
SFCz		-		-	0.16	0.08	36
M-2CzDQ	6	1140	5.6	442	0.15	0.06	42
PPISCN	2.8	-	5.56	-	0.157	0.073	43
C2PPI			5.55	406	0.162	0.058	33
DSiTPI			5.3	406	0.16	0.06	31
2BuCz-CNCz	3.6	2.65	5.24	408	0.157	0.05	44
PHDPYCZ	3.4	4348	5.17	414	0.159	0.056	16
PCZPBO			5.1	414	0.15	0.08	45
3nPI-BP-4PI			4.95		0.15	0.06	46
OMeNPI-PITPA	2.9	13 710	4.9	443	0.15	0.07	47
PI-NP-3-Cz			4.78	410	0.151	0.055	48
CPBPMCN	3.4	10800	4.71	-	0.15	0.08	49
mCzAnN	3.6	5294	4.7	-	0.15	0.06	50
MeNPI-PITPA	3.2	13 599	4.7	441	0.15	0.08	47
SAFpCN			4.63	432	0.153	0.054	51
PIS			4.59		0.152	0.067	52
SAF-PI			4.57	428	0.156	0.053	53
Ph-BPA-BPI			4.56	448	0.15	0.08	54
tPCZDPO			4.5	424	0.16	0.05	55
BCzB-PPI	3.05	11364	4.43	439	0.157	0.08	56
PIPD-MP-DPA	2.9	4.158	4.4	428	0.154	0.078	57
2FPPICz	3	3835	4.3	425	0.16	0.045	58
ATPNF-2			4.3	444	0.15	0.06	32
DFPBI			4.18	435	0.154	0.042	59
6,9-CzPPI			4.14	408	0.157	0.061	60
DSFX-TFB			4.1		0.15	0.08	61
DPM	3.1	-	4	4.28	0.157	0.053	62
3BCzPT	2.8	9155	3.81	-	0.151	0.053	63
PhImAc	4.1	20678	3.68	431	0.15	0.07	64
9-PCZOXZ	3.4	4295	3.6	410	0.1573	0.0761	30
PhAA	3.8	2267	3.6	-	0.15	0.05	65
BDPA			3.55	435	0.153	0.06	66
PPINCN			3.55		0.15	0.08	67
DPACPhTPI	3.3	-	3.51	436	0.16	0.078	68
IDCz-BPSP			3.48		0.154	0.059	69
4,9-DTBP	4.2	-	3.47	433	0.158	0.063	70
TPIBCz			3.38	435	0.154	0.063	71

mTPA-PPI	3.2	4065	3.33	404	0.161	0.049	72
CCsCN	3	6338	3.33	431	0.15	0.08	73
OCI	3	8024	3.19	-	0.156	0.054	74
OCT	3.8	446	3.19		0.156	0.054	74
SAFmCN			3.18	413	0.16	0.046	51
DPACFPPI			3.03	426	0.162	0.057	68
CTpCN	3	8032	3.01	433	0.15	0.08	73
TFSTPA	-	2838	2.7	432	0.16	0.07	75
p-DSiTP	4	1358	2.7		0.162	0.061	76
DCCPPI			2.64	430	0.15	0.06	77
TDPM			2.6		0.158	0.045	62
TPA-2PI			2.6	458	0.14	0.08	78
CNPIS			2.59		0.152	0.064	52
PhImEn	4.3	11696	2.5	414	0.15	0.05	64
DPACTPI	3.6	-	2.31	428	0.165	0.068	68
MPPPI	4.5	3812	2.1	396	0.15	0.07	79
TpXC	3.1	1607	2.1	416	0.155	0.074	80
TPA-PA	-	11970	7.23	428	0.157	0.073	81
BCzPSP			1.95		0.152	0.076	69
PPI	412	3307	1.86	412	0.161	0.065	72
TIP	3.2	-	1.77	428	0.158	0.069	82
BCzb-PIM	3.11	8342	1.72	437	0.159	0.075	56
TmPC			1.72	426	0.154	0.066	80
PI-NP-TPA	-	-	1.51	420	0.151	0.072	48
MPPIS-Cz	5.2	4801	1.48	430	0.16	0.08	83
SBF-PI-SPF	3.5	3775	6.19	436	0.155	0.049	35

Table S8. Cartesian coordinates for the optimized structure of S_0 state geometry of *p*TCN.

N	−6.21635	0.12469	0.02919
C	−4.8151	−0.03738	−0.01586
C	−4.16121	−0.87999	0.90207
H	−4.74503	−1.39509	1.66548
C	−2.77985	−1.04916	0.84732
H	−2.29147	−1.69226	1.58161
C	−1.99774	−0.37309	−0.10814
C	−2.65907	0.47867	−1.01064
H	−2.07689	0.99976	−1.77326
C	−4.04332	0.6393	−0.97658
H	−4.53706	1.28679	−1.70177
C	−0.52023	−0.50031	−0.13535
C	0.13178	−1.78483	−0.23766
C	−0.60623	−2.98314	−0.42816
H	−1.69048	−2.92699	−0.51338
C	0.02705	−4.20824	−0.52537
H	−0.55974	−5.11615	−0.67598
C	1.43217	−4.27887	−0.44175
H	1.93755	−5.24335	−0.51705
C	2.17672	−3.12403	−0.28208
H	3.26154	−3.20629	−0.24616
C	1.5606	−1.84888	−0.18427
C	2.3355	−0.6276	−0.06015
C	3.75742	−0.65169	0.0554
H	4.27748	−1.6068	0.10059
C	4.51901	0.48942	0.16402
C	3.87567	1.78038	0.12161
C	4.62605	2.98592	0.12671
H	5.7138	2.93133	0.15254
C	4.00128	4.21848	0.08125
H	4.59657	5.13328	0.08243
C	2.59488	4.28865	0.02029
H	2.09655	5.259	−0.01748
C	1.84179	3.12843	−0.00924
H	0.75895	3.21336	−0.08148
C	2.44927	1.84629	0.03321
C	1.67192	0.62173	−0.036
C	0.24609	0.6414	−0.06065
H	−0.27817	1.59149	0.02482

C	5.98907	0.36037	0.3279
C	6.74318	−0.34375	−0.59763
H	6.25552	−0.76422	−1.48023
C	8.14479	−0.51749	−0.43971
C	8.92881	−1.23152	−1.38812
H	8.43494	−1.65883	−2.26368
C	10.28861	−1.38329	−1.20818
H	10.87882	−1.93372	−1.94309
C	10.9268	−0.82654	−0.07095
H	12.00283	−0.95341	0.0609
C	10.19301	−0.12685	0.86573
H	10.68159	0.30352	1.7428
C	8.79105	0.04715	0.7081
C	8.00016	0.76104	1.65111
H	8.48586	1.18176	2.53459
C	6.64347	0.91546	1.46832
H	6.04905	1.4525	2.20939
C	−6.79221	1.39514	−0.20863
C	−7.95732	1.51394	−0.98636
H	−8.40949	0.61762	−1.41192
C	−8.52829	2.76797	−1.20905
H	−9.43317	2.844	−1.81497
C	−7.94086	3.92167	−0.67908
H	−8.386	4.90082	−0.86143
C	−6.77655	3.80588	0.08788
H	−6.31201	4.69687	0.51463
C	−6.20869	2.55452	0.33191
H	−5.30978	2.46545	0.94269
C	−7.04948	−0.98446	0.30859
C	−6.7794	−2.24044	−0.26265
H	−5.92456	−2.35099	−0.93055
C	−7.59912	−3.33323	0.02352
H	−7.3765	−4.30211	−0.42758
C	−8.70818	−3.18985	0.86411
H	−9.35088	−4.04461	1.07968
C	−8.98434	−1.93824	1.42472
H	−9.8426	−1.81342	2.08771
C	−8.1596	−0.84387	1.15964
H	−8.3689	0.12744	1.60877

Table S9. Cartesian coordinates for the optimized structure of S_0 state geometry of *m*TCN.

N	5.49913	−0.33485	0.02739
C	4.61154	0.59849	0.61453
C	3.24848	0.58577	0.2713
H	2.8934	−0.12874	−0.47225
C	2.33887	1.4776	0.85959
C	2.81609	2.41536	1.79526
H	2.12011	3.10163	2.27958
C	4.17404	2.44474	2.12332
H	4.53685	3.16492	2.8596
C	5.07198	1.54296	1.54849
H	6.12695	1.56021	1.82395
C	0.89257	1.37418	0.52562
C	0.12263	2.50729	0.06724
C	0.72752	3.76511	−0.1992
H	1.80364	3.87155	−0.06868
C	−0.01958	4.8443	−0.63417
H	0.46758	5.79994	−0.83678
C	−1.40909	4.70306	−0.82334
H	−2.00539	5.55269	−1.16128
C	−2.01954	3.48231	−0.59701
H	−3.09109	3.3993	−0.77019
C	−1.28252	2.34894	−0.16155
C	−1.90749	1.0515	0.03303
C	−3.30443	0.85138	−0.18372
H	−3.92274	1.68492	−0.5122
C	−3.92087	−0.37013	−0.02942
C	−3.14144	−1.51241	0.38695
C	−3.74216	−2.77728	0.62715
H	−4.8208	−2.88088	0.51266
C	−2.98581	−3.86865	1.01336
H	−3.46784	−4.83116	1.19455
C	−1.59226	−3.73219	1.1754
H	−0.98826	−4.59328	1.4677
C	−0.98574	−2.50481	0.97421
H	0.09179	−2.43045	1.11178
C	−1.73327	−1.35879	0.59318
C	−1.11515	−0.05489	0.42123
C	0.27494	0.1512	0.6674
H	0.88435	−0.67895	1.02023

C	6.79034	0.07078	−0.38779
C	6.98161	1.31678	−1.01295
H	6.12325	1.96725	−1.1846
C	8.25852	1.71598	−1.41206
H	8.38843	2.68606	−1.89609
C	9.36135	0.87807	−1.21461
H	10.35654	1.19052	−1.5347
C	9.17251	−0.36557	−0.60221
H	10.02443	−1.02767	−0.43473
C	7.90268	−0.76618	−0.18248
H	7.76424	−1.73031	0.30803
C	5.08487	−1.67899	−0.14548
C	4.37779	−2.34238	0.87443
H	4.1648	−1.81653	1.80605
C	3.95627	−3.66158	0.69624
H	3.41151	−4.16286	1.49905
C	4.24678	−4.34751	−0.48839
H	3.92167	−5.38042	−0.62204
C	4.96017	−3.69407	−1.49895
H	5.18822	−4.2146	−2.43122
C	5.37017	−2.36899	−1.33727
H	5.91248	−1.85883	−2.13422
C	−5.37765	−0.47963	−0.30958
C	−6.28922	0.31282	0.37163
H	−5.93571	0.98675	1.15581
C	−7.68218	0.25959	0.09062
C	−8.6257	1.06312	0.79055
H	−8.26581	1.74141	1.56767
C	−9.97213	0.98947	0.49572
H	−10.68536	1.61148	1.03994
C	−10.43708	0.1076	−0.51296
H	−11.50421	0.05828	−0.73769
C	−9.54643	−0.68577	−1.20818
H	−9.90178	−1.3662	−1.98555
C	−8.15338	−0.63333	−0.927
C	−7.20327	−1.43418	−1.62095
H	−7.55484	−2.10683	−2.40697
C	−5.85968	−1.36301	−1.32257
H	−5.14447	−1.97357	−1.87679

References

1. Dolomanov OV, Bourhis LJ, Gildea RJ *et al. OLEX2* : a complete structure solution, refinement and analysis program. *J Appl Crystallogr* 2009; **42**: 339–41.
2. Sheldrick GM. *SHELXT* – Integrated space-group and crystal-structure determination. *Acta Crystallogr Sect Found Adv* 2015; **71**: 3–8.
3. Sheldrick GM. Crystal structure refinement with *SHELXL*. *Acta Crystallogr Sect C Struct Chem* 2015; **71**: 3–8.
4. Frisch MJ, Trucks GW, Schlegel HB *et al.* Gaussian 16 Revision B.01. 2016.
5. Neese F. Software update: The ORCA program system—Version 5.0. *WIREs Comput Mol Sci* 2022; **12**: e1606.
6. Hellweg A, Hättig C, Höfener S *et al.* Optimized accurate auxiliary basis sets for RI-MP2 and RI-CC2 calculations for the atoms Rb to Rn. *Theor Chem Acc* 2007; **117**: 587–97.
7. Weigend F, Ahlrichs R. Balanced basis sets of split valence, triple zeta valence and quadruple zeta valence quality for H to Rn: Design and assessment of accuracy. *Phys Chem Chem Phys* 2005; **7**: 3297.
8. Weigend F. Accurate Coulomb-fitting basis sets for H to Rn. *Phys Chem Chem Phys* 2006; **8**: 1057.
9. Humphrey W, Dalke A, Schulten K. VMD: Visual molecular dynamics. *J Mol Graph* 1996; **14**: 33–8.
10. Liu Z, Lu T, Chen Q. An sp-hybridized all-carboatomic ring, cyclo[18]carbon: Electronic structure, electronic spectrum, and optical nonlinearity. *Carbon* 2020; **165**: 461–7.
11. Lu T, Chen Q. Interaction region indicator: A simple real space function clearly revealing both chemical bonds and weak interactions**. *Chemistry–Methods* 2021; **1**: 231–9.
12. Chance RR, Prock A, Silbey R. Molecular fluorescence and energy transfer near interfaces. In: Prigogine I, Rice SA (eds.). *Advances in Chemical Physics*. New York: Wiley, 1978, 1–65.
13. Du C, Liu H, Cheng Z *et al.* Ultraefficient non-doped deep blue fluorescent OLED: Achieving a high EQE of 10.17% at 1000 cd m^{-2} with CIE_y <0.08. *Adv Funct Mater* 2023; **33**: 2304854.
14. Xiao S, Gao Y, Wang R *et al.* Highly efficient hybridized local and charge-transfer (HLCT) deep-blue electroluminescence with excellent molecular horizontal orientation. *Chem Eng J* 2022; **440**: 135911.
15. Guo R, Liu W, Ying S *et al.* Exceptionally efficient deep blue anthracene-based luminogens: design, synthesis, photophysical, and electroluminescent mechanisms. *Sci Bull* 2021; **66**: 2090–8.

16. Ma B, Zhang B, Zhang H *et al.* Asymmetric structural engineering of hot-exciton emitters achieving a breakthrough in non-doped BT.2020 blue OLEDs with a record 9.5% external quantum efficiency. *Adv Sci* 2024; **11**: 2407254.
17. Liao C, Chen B, Xie Q *et al.* A breakthrough in solution-processed ultra-deep-blue HLCT OLEDs: A record external quantum efficiency exceeding 10% based on novel v-shaped emitters. *Adv Mater* 2023; **35**: 2305310.
18. Jayabharathi J, Thilagavathy S, Thanikachalam V *et al.* A triphenylacrylonitrile phenanthroimidazole cored butterfly shaped AIE chromophore for blue and HLCT sensitized fluorescent OLEDs. *J Mater Chem C* 2022; **10**: 4342–54.
19. Huh J-S, Ha YH, Kwon S-K *et al.* Design strategy of anthracene-based fluorophores toward high-efficiency deep blue organic light-emitting diodes utilizing triplet–triplet fusion. *ACS Appl Mater Interfaces* 2020; **12**: 15422–9.
20. Li Z, Li C, Xu Y *et al.* Nonsymmetrical connection of two identical building blocks: constructing donor–acceptor molecules as deep blue emitting materials for efficient organic emitting diodes. *J Phys Chem Lett* 2019; **10**: 842–7.
21. Ge S, Du C, Cheng Z *et al.* Highly efficient non-doped organic light emitting diodes based on phenanthreneimidazole derivatives with hot exciton mechanism. *Chem Eng J* 2024; **489**: 151314.
22. Du C, Lu T, Cheng Z *et al.* Rational molecular design of phenanthroimidazole-based fluorescent materials towards high-efficiency non-doped deep blue OLEDs. *J Mater Chem C* 2022; **10**: 14186–93.
23. Li C, Wang S, Chen W *et al.* High performance full color OLEDs based on a class of molecules with dual carrier transport channels and small singlet–triplet splitting. *Chem Commun* 2015; **51**: 10632–5.
24. Liu C, Zhou Y, Li T *et al.* Efficient nondoped Deep-Blue electrofluorescence benefiting from structural hindrance and regular C-H···π stacking. *Chem Eng J* 2023; **471**: 144505.
25. Lee H, Patil VV, Lim J *et al.* Nearly 100% exciton utilization via hybridized inter- and intramolecular triplet exciton harvesting channels in blue fluorescent organic light-emitting diodes. *Adv Opt Mater* 2022; **10**: 2200256.
26. Xu L, Sun M, Zhou Y *et al.* A new multifunctional fluorescent molecule for highly efficient non-doped deep-blue electro-fluorescence with high color-purity and efficient phosphorescent OLEDs. *Org Chem Front* 2023; **10**: 490–8.
27. Xu P, Xu L, Pan Y *et al.* Novel deep-blue hot exciton material for high-efficiency nondoped organic light-emitting diodes. *J Mater Chem C* 2022; **10**: 6596–602.

28. Han P, Xu Z, Lin C *et al.* Tetraphenylbenzene-based AIEgens: horizontally oriented emitters for highly efficient non-doped deep blue OLEDs and hosts for high-performance hybrid WOLEDs. *J Mater Chem C* 2020; **8**: 7012–8.
29. Liu B, Yu Z-W, He D *et al.* Ambipolar D–A type bifunctional materials with hybridized local and charge-transfer excited state for high performance electroluminescence with EQE of 7.20% and CIEy ~ 0.06. *J Mater Chem C* 2017; **5**: 5402–10.
30. Wang R, Li T, Liu C *et al.* Efficient non-doped blue electro-fluorescence with boosted and balanced carrier mobilities. *Adv Funct Mater* 2022; **32**: 2201143.
31. Zheng Y, Zhu X, Ni Z *et al.* Bipolar molecules with hybridized local and charge-transfer state for highly efficient deep-blue organic light-emitting diodes with EQE of 7.4% and CIE_y ~ 0.05. *Adv Opt Mater* 2021; **9**: 2100965.
32. Jeong Y, Hwang KM, Lee JH *et al.* Asymmetric anthracene hosts decorated with naphthobenzofurocarbazole for highly efficient deep-blue organic light-emitting diodes and low-efficiency roll-off. *J Mater Chem C* 2023; **11**: 10911–22.
33. Zhong Z, Liu Z, Geng S *et al.* Highly twisted bipolar molecules for efficient near-ultraviolet organic light-emitting diodes *via* a hybridized local and charge-transfer mechanism. *J Mater Chem C* 2023; **11**: 1733–41.
34. Malatong R, Waengdongbung W, Nalaoh P *et al.* Deep-Blue triplet–triplet annihilation organic light-emitting diode (ciey ≈ 0.05) using tetraphenylimidazole and benzonitrile functionalized anthracene/chrysene emitters. *Molecules* 2022; **27**: 8923.
35. Tang S, Yang G, Zhu J *et al.* Multifunctional materials serving as efficient non-doped violet-blue emitters and host materials for phosphorescence. *Chem – Eur J* 2021; **27**: 9102–11.
36. Zhen Y, Zhang F, Liu H *et al.* Impact of peripheral groups on pyrimidine acceptor-based HLCT materials for efficient deep blue OLED devices. *J Mater Chem C* 2022; **10**: 9953–60.
37. Li Z, Xie N, Xu Y *et al.* Fluorine-substituted phenanthro[9,10-d]imidazole derivatives with optimized charge-transfer characteristics for efficient deep-blue emitters. *Org Mater* 2020; **02**: 011–9.
38. Wang X, Li X, Yang R *et al.* Simple and efficient non-doped deep-blue and white organic light-emitting diode based on hybridized local and charge transfer (HLCT) materials. *New J Chem* 2024; **48**: 10262–72.
39. Jia Y, Zhang Y, Fan S *et al.* A novel bipolar carbazole/ phenanthroimidazole derivative for high efficiency nondoped deep-blue organic light-emitting diodes. *Org Electron* 2019; **64**: 259–65.

40. Zhu J-J, Chen W-C, Yuan Y *et al.* Rational molecular design of bipolar phenanthroimidazole derivatives to realize highly efficient non-doped deep blue electroluminescence with CIEy < 0.06 and EQE approaching 6%. *Dyes Pigments* 2020; **173**: 107982.
41. Li G, Li B, Zhang H *et al.* Efficient ultraviolet organic light-emitting diodes with a CIEy of 0.04 and negligible-efficiency roll-off. *ACS Appl Mater Interfaces* 2022; **14**: 10627–36.
42. Wu G, Ge X, Yang Z *et al.* High-efficiency blue hybridized local and charge-transfer fluorescent material affording OLEDs with external quantum efficiency exceeding 14 %. *Chem Eng J* 2024; **497**: 154659.
43. Lv J, Song S, Li J *et al.* High and balanced bipolar-transporting deep-blue HLCT material for efficient monochrome and white OLEDs based on a simple phenanthroimidazole-dibenzothiophene derivative. *Adv Opt Mater* 2024; **12**: 2301413.
44. Zhang H, Li G, Guo X *et al.* High-performance ultraviolet organic light-emitting diode enabled by high-lying reverse intersystem crossing. *Angew Chem Int Ed* 2021; **60**: 22241–7.
45. Xie M, Li T, Liu C *et al.* Realizing highly efficient blue electrofluorescence by optimized hybridized local and charge transfer state and balanced carrier mobilities. *Chem Eng J* 2023; **472**: 144950.
46. Chen M, Yuan Y, Zheng J *et al.* Novel bipolar phenanthroimidazole derivative design for a nondoped deep-blue emitter with high singlet exciton yields. *Adv Opt Mater* 2015; **3**: 1215–9.
47. Jayabharathi J, Sujatha P, Thanikachalam V *et al.* Efficient non-doped blue organic light-emitting diodes: donor–acceptor type host materials. *RSC Adv* 2017; **7**: 54078–86.
48. Liu Y, Tao T, Hu H-C *et al.* Fine regulation of linker and donor moieties to construct benzimidazole-based blue emitters for high-efficient organic light-emitting diodes. *Dyes Pigments* 2021; **188**: 109191.
49. Tan Y, Zhao Z, Shang L *et al.* A novel bipolar D–π–A type phenanthroimidazole/carbazole hybrid material for high efficiency nondoped deep-blue organic light-emitting diodes with NTSC CIE_y and low efficiency roll-off. *J Mater Chem C* 2017; **5**: 11901–9.
50. Wang Z, Yang T, Dong S *et al.* Anthracene and carbazole based asymmetric fluorescent materials for high-efficiency deep-blue non-doped organic light emitting devices with CIEy=0.06. *Dyes Pigments* 2022; **199**: 110047.
51. Sudyoadsuk T, Petdee S, Kaiyasuan C *et al.* Cyanophenyl spiro[acridine-9,9′-fluorene]s as simple structured hybridized local and charge-transfer-based ultra-deep blue emitters for highly efficient non-doped electroluminescent devices (CIE*y* ≤ 0.05). *J Mater Chem C* 2021; **9**: 6251–6.

52. Lv J, Huo Y, Xiao S *et al.* Regulation of excited-state properties of dibenzothiophene-based fluorophores for realizing efficient deep-blue and HLCT-sensitized OLEDs. *Mater Chem Front* 2023; **7**: 85–95.
53. Kaiyasuan C, Chasing P, Nalaoh P *et al.* Twisted phenanthro[9,10-d]imidazole derivatives as non-doped emitters for efficient electroluminescent devices with ultra-deep blue emission and high exciton utilization efficiency. *Chem – Asian J* 2021; **16**: 2328–37.
54. Liu B, Yuan Y, He D *et al.* High-performance blue OLEDs based on phenanthroimidazole emitters via substitutions at the C6- and C9-positions for improving exciton utilization. *Chem – Eur J* 2016; **22**: 12130–7.
55. Sun M, Ma C, Xie M *et al.* An efficient blue electro-fluorescence material with high electron and balanced carrier mobilities based on effective π-stacking between acceptors. *Org Chem Front* 2023; **10**: 4878–86.
56. Yu P, Xiao Y. Non-doped deep-blue OLEDs based on carbazole-π-imidazole derivatives. *Materials* 2021; **14**: 2349.
57. Xu J, Liu H, Li J *et al.* Multifunctional bipolar materials serving as emitters for efficient deep-blue fluorescent OLEDs and as hosts for phosphorescent and white OLEDs. *Adv Opt Mater* 2021; **9**: 2001840.
58. Xin J, Li Z, Liu Y *et al.* High-efficiency non-doped deep-blue fluorescent organic light-emitting diodes based on carbazole/phenanthroimidazole derivatives. *J Mater Chem C* 2020; **8**: 10185–90.
59. Qiu X, Ying S, Wang C *et al.* Novel 9,9-dimethylfluorene-bridged D–π–A-type fluorophores with a hybridized local and charge-transfer excited state for deep-blue electroluminescence with $CIE_y \sim 0.05$. *J Mater Chem C* 2019; **7**: 592–600.
60. Wang X, Liu Z, Geng S *et al.* Donor–acceptor–donor molecules for high performance near ultraviolet organic light-emitting diodes *via* hybridized local and charge-transfer processes. *J Mater Chem C* 2023; **11**: 5316–23.
61. Sun M-L, Zhu W-S, Zhang Z-S *et al.* Nondoped deep-blue spirofluorenexanthene-based green organic semiconductors (GOS) via a pot, atom and step economic (PASE) route combining direct arylation with tandem reaction. *J Mater Chem C* 2015; **3**: 94–9.
62. Xiao S, Zhang S-T, Gao Y *et al.* Efficient and stable deep-blue narrow-spectrum electroluminescence based on hybridized local and charge-transfer (HLCT) state. *Dyes Pigments* 2021; **193**: 109482.

63. Wang J, Yang Y, Yao C *et al.* Aggregation-induced emission phenanthroline derivatives for ultrahigh color purity deep blue OLEDs with CIEx≤0.15 and CIEy≤0.08 and low efficiency roll-off. *J Lumin* 2023; **259**: 119829.
64. Chen S, Lian J, Wang W *et al.* Efficient deep blue electroluminescence with CIE $y \in (0.05–0.07)$ from phenanthroimidazole–acridine derivative hybrid fluorophores. *J Mater Chem C* 2018; **6**: 9363–73.
65. Huang Y, Du X, Tao S *et al.* High efficiency non-doped deep-blue and fluorescent/phosphorescent white organic light-emitting diodes based on an anthracene derivative. *Synth Met* 2015; **203**: 49–53.
66. Park H, Lee J, Kang I *et al.* Highly rigid and twisted anthracene derivatives: a strategy for deep blue OLED materials with theoretical limit efficiency. *J Mater Chem* 2012; **22**: 2695–700.
67. Jayabharathi J, Anudeebhana J, Thanikachalam V *et al.* Multifunctional assistant acceptor modulated pyrenyl phenanthrimidazole derivatives for highly efficient blue and host-sensitized OLEDs. *J Mater Chem C* 2021; **9**: 15683–97.
68. Huang Z, Wang B, Zhang Q *et al.* Highly twisted bipolar emitter for efficient nondoped deep-blue electroluminescence. *Dyes Pigments* 2017; **140**: 328–36.
69. Wang J, Zhai X, Ji C *et al.* Simple A–D–A pure blue fluorescent emitters based on hybridized local and charge-transfer excited state for non-doped OLEDs with narrow full width at half-maximum of 0.20 eV and CIEy< 0.06. *Dyes Pigments* 2023; **219**: 111586.
70. Jung M, Lee J, Jung H *et al.* Highly efficient pyrene blue emitters for OLEDs based on substitution position effect. *Dyes Pigments* 2018; **158**: 42–9.
71. Chen W-C, Yuan Y, Ni S-F *et al.* Achieving efficient violet-blue electroluminescence with CIE_y 6% from naphthyl-linked phenanthroimidazole–carbazole hybrid fluorophores. *Chem Sci* 2017; **8**: 3599–608.
72. Liu H, Bai Q, Yao L *et al.* Highly efficient near ultraviolet organic light-emitting diode based on a meta-linked donor–acceptor molecule. *Chem Sci* 2015; **6**: 3797–804.
73. Chantanop N, Nalaoh P, Chasing P *et al.* Chrysene and triphenylene based-fluorophores as non-doped deep blue emitters for triplet-triplet annihilation organic light-emitting diodes. *J Lumin* 2022; **248**: 118926.
74. Zhou H, Yin M, Zhao Z *et al.* Novel carbazole-based multifunctional materials with a hybridized local and charge-transfer excited state acting as deep-blue emitters and phosphorescent hosts for highly efficient organic light-emitting diodes. *J Mater Chem C* 2021; **9**: 5899–907.

75. Jiang Z, Liu Z, Yang C *et al.* Multifunctional fluorene-based oligomers with novel spiro-annulated triarylamine: efficient, stable deep-blue electroluminescence, good hole injection, and transporting materials with very high T_g. *Adv Funct Mater* 2009; **19**: 3987–95.
76. Shen Z, Zhu X, Tang W *et al.* Twisted donor–acceptor molecules for efficient deep blue electroluminescence with CIE_y ~ 0.06. *J Mater Chem C* 2020; **8**: 9401–9.
77. Huo J, Gao C, Cao Y *et al.* Rational design of phenanthroimidazole derivatives with hybridized local and charge-transfer characteristics to achieve efficient blue emission in non-doped OLEDs. *J Mater Chem C* 2023; **11**: 4456–65.
78. Yu Y, Zhao R, Liu H *et al.* Highly efficient deep-blue light-emitting material based on V-Shaped donor-acceptor triphenylamine-phenanthro[9,10-d]imidazole molecule. *Dyes Pigments* 2020; **180**: 108511.
79. Thanikachalam V, Jeeva P, Jayabharathi J. Hybridised-local and charge-transfer excited states in donor-spacer-acceptor molecules for efficient OLEDs: Combined experimental and theoretical study. *ChemistrySelect* 2017; **2**: 1860–70.
80. Chawanpunyawat T, Chasing P, Nalaoh P *et al.* Rational design of chrysene-based hybridized local and charge-transfer molecules as efficient non-doped deep-blue emitters for simple-structured electroluminescent devices. *Chem – Asian J* 2021; **16**: 4145–54.
81. Chawanpunyawat T, Chasing P, Nalaoh P *et al.* Rational design of chrysene-based hybridized local and charge-transfer molecules as efficient non-doped deep-blue emitters for simple-structured electroluminescent devices. *Chem – Asian J* 2021; **16**: 4145–54.
82. Jeong S, Hong J-I. Extremely deep-blue fluorescent emitters with CIEy ≤ 0.04 for non-doped organic light-emitting diodes based on an indenophenanthrene core. *Dyes Pigments* 2017; **144**: 9–16.
83. Thanikachalam V, Jeeva P, Jayabharathi J. Highly efficient non-doped blue organic light emitting diodes based on a D–π–A chromophore with different donor moieties. *RSC Adv* 2017; **7**: 13604–14.
